# Loss of CAPS2/Cadps2 leads to exocrine pancreatic cell injury and intracellular accumulation of secretory granules in mice

**DOI:** 10.3389/fmolb.2022.1040237

**Published:** 2022-11-07

**Authors:** Yotaroh Sato, Miho Tsuyusaki, Hiromi Takahashi-Iwanaga, Rena Fujisawa, Atsushi Masamune, Shin Hamada, Ryotaro Matsumoto, Yu Tanaka, Yoichi Kakuta, Yumi Yamaguchi-Kabata, Tamio Furuse, Shigeharu Wakana, Takuya Shimura, Rika Kobayashi, Yo Shinoda, Ryo Goitsuka, So Maezawa, Tetsushi Sadakata, Yoshitake Sano, Teiichi Furuichi

**Affiliations:** ^1^ Department of Applied Biological Science, Faculty of Science and Technology, Tokyo University of Science, Chiba, Japan; ^2^ Laboratory of Histology and Cytology, Graduate School of Medicine, Hokkaido University, Sapporo, Japan; ^3^ Division of Gastroenterology, Tohoku University Graduate School of Medicine, Sendai, Japan; ^4^ Group of Materials and Information Management, Tohoku Medical Megabank Organization, Tohoku University, Sendai, Japan; ^5^ Technology and Development Team for Mouse Phenotype Analysis, RIKEN BioResource Research Center, Ibaraki, Japan; ^6^ Department of Internal Medicine (Endocrinology and Metabolism), Faculty of Medicine, University of Tsukuba, Tsukuba, Japan; ^7^ Department of Environmental Health, School of Pharmacy, Tokyo University of Pharmacy and Life Sciences, Hachioji, Japan; ^8^ Division of Cell Fate Regulation, Research Institute for Biomedical Sciences, Tokyo University of Science, Noda, Japan; ^9^ Education and Research Support Center, Gunma University Graduate School of Medicine, Maebashi, Gunma, Japan

**Keywords:** pancreatic acinar cells, *Cadps2*, secretory granules, amylase, exocrine pancreas, CAPS2

## Abstract

The type 2 Ca^2+^-dependent activator protein for secretion (CAPS2/CADPS2) regulates dense-core vesicle trafficking and exocytosis and is involved in the regulated release of catecholamines, peptidergic hormones, and neuromodulators. CAPS2 is expressed in the pancreatic exocrine acinar cells that produce and secrete digestive enzymes. However, the functional role of CAPS2 in vesicular trafficking and/or exocytosis of non-regulatory proteins in the exocrine pancreas remains to be determined. Here, we analyzed the morpho-pathological indicators of the pancreatic exocrine pathway in *Cadps2*-deficient mouse models using histochemistry, biochemistry, and electron microscopy. We used whole exosome sequencing to identify *CADPS2* variants in patients with chronic pancreatitis (CP). *Caps2/Cadps2*-knockout (KO) mice exhibited morphophysiological abnormalities in the exocrine pancreas, including excessive accumulation of secretory granules (zymogen granules) and their amylase content in the cytoplasm, deterioration of the fine intracellular membrane structures (disorganized rough endoplasmic reticulum, dilated Golgi cisternae, and the appearance of empty vesicles and autophagic-like vacuoles), as well as exocrine pancreatic cell injury, including acinar cell atrophy, increased fibrosis, and inflammatory cell infiltration. Pancreas-specific *Cadps2* conditional KO mice exhibited pathological abnormalities in the exocrine pancreas similar to the global *Cadps2* KO mice, indicating that these phenotypes were caused either directly or indirectly by CAPS2 deficiency in the pancreas. Furthermore, we identified a rare variant in the exon3 coding region of *CADPS2* in a non-alcoholic patient with CP and showed that *Cadps2-dex3* mice lacking CAPS2 exon3 exhibited symptoms similar to those exhibited by the *Cadps2* KO and cKO mice. These results suggest that CAPS2 is critical for the proper functioning of the pancreatic exocrine pathway, and its deficiency is associated with a risk of pancreatic acinar cell pathology.

## Introduction

The Ca^2+^-dependent activator protein for secretion (CAPS/CADPS) protein family consists of two cytosolic protein members (CAPS1/CADPS1 and CAPS2/CADPS2) that play a pivotal role in the regulated exocytosis of dense-core vesicles (DCVs) in neuronal and neuroendocrine cells (Hay and Martin, 1992; Martin and Kowalchyk, 1997; Berwin et al., 1998; Speidel et al., 2003; Sadakata et al., 2004; Sadakata et al., 2006; Khodthong et al., 2011). We previously showed that CAPS2 promoted the release of the neurotrophins brain-derived neurotrophic factor (BDNF) and neurotrrophin-3 (Sadakata et al., 2004; Shinoda et al., 2011), as well as the neuropeptides pro-opiomelanocortin (Fujima et al., 2020) and oxytocin (Fujima et al., 2021). We also demonstrated the involvement of CAPS2 in membrane trafficking as part of the *trans*-Golgi network (TGN) (Sadakata et al., 2012a). *Cadps2* knockout (KO) mice do not exhibit obvious differences in life expectancy compared to wild-type (WT) mice under standard breeding conditions (Sadakata et al., 2007a; Sadakata et al., 2007b). However, *Cadps2* KO mice exhibit smaller body size and lower body weight throughout the postnatal period compared to WT mice despite slightly increased food consumption (per body weight) (Sadakata et al., 2007b; Mishima et al., 2015).

We hypothesized that these phenotypes in *Cadps2* KO mice could be due to nutrition-, digestion-, and/or metabolism-related issues. Importantly, CAPS2 is the predominant CAPS family protein expressed in the pancreatic exocrine acinar cells (Sadakata et al., 2007c). Pancreatic acini produce and secrete digestive enzymes that are packaged in large DCVs or secretory granules (SGs; also called zymogen granules). However, whether CAPS2 affects the trafficking and/or exocytosis of SGs in pancreatic acinar cells remains to be determined.

Here, we investigated the role of CAPS2 in pancreatic exocrine function by analyzing the morphological and pathophysiological indicators of the pancreatic exocrine pathway in *Cadps2* global KO mice, as well as either pancreas-specific *Cadps2* conditional KO (cKO) mice or *Cadps2-dex3* mice expressing a subcellular localization-defective *Cadps2* variant (Sadakata et al., 2007a; Sadakata et al., 2012b). Finally, we analyzed variations of *CADPS2* in patients with chronic pancreatitis (CP) *via* whole exosome sequencing.

## Materials and methods

### Mice

This study used the *Cadps2*-KO (*Cadps2* ^*−/−*^ or *Cadps2* ^*tm1Tfr*^ */Cadps2* ^*tm1Tfr*^) (Mouse Genome Informatics [MGI] ID: 3707315) and the corresponding WT control (*Cadps2* ^*+/+*^) mice (Sadakata et al., 2007a; Sadakata et al., 2007b). *Ptf1a-Cre* (*Ptf1a* ^*tm1(cre)Cvw*^) mice (Kawaguchi et al., 2002; Sadakata et al., 2013) (MGI ID: 2387804) were kindly provided by Prof. Christopher V.E. Wright of Vanderbilt University. We mated *Cadps2* ^*flox/flox*^ (or *Cadps2* ^*tm1.1Ksak/tm1.1Ksak*^) female mice (Fujima et al., 2021) with *Cadps2* ^*flox/+*^ *Ptf1a* ^*cre/+*^ male mice (Sadakata et al., 2013) to produce *Cadps2* ^*flox/flox*^ *Ptf1a* ^*+/+*^ as a control and *Cadps2* ^*flox/flo*x^ *Ptf1a* ^*cre/+*^ as *Cadps2* cKO mice. In addition, the *Cadps2-dex3* (referred in short as *dex3*) mice (*Cadps2* ^*tm2Tfr*^ */Cadps2* ^*tm2Tfr*^) (Sadakata et al., 2012b) (MGI ID: 5705653) were also used. All genetically manipulated *Cadps2* mice were generated by our group. Mice were housed in a group at the Tokyo University of Science in sterile containers at ambient temperature within a pathogen-free barrier facility under a 12 h light/12 h dark cycle, with access to free water and standard rodent chow. All animal procedures were approved by the Tokyo University of Science Animal Care and Use Committee (approval reference No. N18003/4, N19004/5, and N20004/5), and were performed in compliance with the ARRIVE guidelines. All experiments were conducted under the Regulations for Animal Research of the Tokyo University of Science.

### Human studies

The exosome sequencing study included 186 Japanese patients with non-alcoholic CP [104 males and 82 females; the median age of onset of 20 years (range 1–77 years)], with 17 cases having a family history of recurrent pancreatitis or CP. This study was approved by the Ethics Committee of Tohoku University Graduate School of Medicine (article#: 2020-1-1105), and all experiments were performed in accordance with the ethical standards of the Declaration of Helsinki. All participants gave written informed consent.

### Antibodies

The following primary antibodies were used: Guinea pig anti-CAPS2 [1:100 (Sadakata et al., 2004; Sadakata et al., 2006)], rabbit anti-α-amylase (1:200; A8273, Sigma-Aldrich, Saint Louis, MO, United States), mouse anti-actin, α-smooth muscle-Cy3 (1:500; C6198, Sigma-Aldrich), goat anti-calnexin (1:100; sc-6465, Santa Cruz Biotechnology, Santa Cruz, CA, United States), mouse anti-GM130 (1:150; 610822, BD Transduction Laboratories, San Jose, CA, United States), mouse APC anti-mouse CD3ε (1:300; 100312, BioLegend Inc., San Diego, CA, United States), and rabbit anti-TGN38 (1:100; T9826, Sigma-Aldrich). The secondary antibodies used were: Alexa Flour 488 goat anti-guinea pig IgG (H + L) (1:500; A11073, Invitrogen, Carlsbad, CA, United States), Alexa Flour 488 goat anti-mouse IgG (H + L) (1:1,000; A28175, Invitrogen), Alexa Flour 488 donkey anti-rabbit IgG (H + L) (1:5,000; A21206, Invitrogen), Alexa Flour 488 donkey anti-mouse IgG (H + L) (1:1,000; A21202, Invitrogen), Alexa Flour 488 donkey anti-mouse IgG (H + L) (1:1,000; A21202, Invitrogen), Alexa Flour 546 donkey anti-goat IgG (H + L) (1:1,000; A11058, Invitrogen), Alexa Fluro555 donkey anti-goat IgG (H + L) (1:1,000; A11055, Invitrogen), and Alexa Flour 594 donkey anti-goat IgG (H + L) (1:1,000; A11058, Invitrogen).

### Preparation of paraffin sections

Mice were deeply anesthetized using pentobarbital sodium (50 mg/kg body weight, intraperitoneal injection, Somnopentyl, Kyoritsu Seiyaku, Tokyo, Japan) and perfused transcardially with saline followed by 4% paraformaldehyde (PFA; 26126-25, Nacali Tesque Inc., Kyoto, Japan) in phosphate-buffered saline (PBS). The pancreas was dissected, cut into small pieces, and post-fixed in 4% PFA overnight at 4°C. Post-fixed specimens were dehydrated and then embedded in a paraffin block. The paraffin block was sectioned at 5 µm thickness on a microtome (SM 2000R, Leica Microsystems, Wetzler, Germany). The sections were used after deparaffinization and rehydration.

### Preparation of frozen sections

The pancreas was dissected from perfused animals as described above, post-fixed in 4% PFA overnight, and subsequently immersed in 30% sucrose in PBS for cryoprotection. Following the fixation and cryoprotection, tissues were embedded in the Tissue-Tek O.C.T compound (4,583, Sakura Finetek, Tokyo, Japan) and frozen using a frozen tissue block preparation apparatus (Histo-Tek PINO-600, Sakura Finetek). Frozen blocks were sectioned at a thickness of 30 µm using a cryostat (CM 1850; Leica Microsystems).

### Immunohistochemistry

Paraffin sections were pretreated by autoclaving at 121°C for 20 min for antigen retrieval. Either paraffin or frozen sections were washed using PBST [0.5% (v/v) Triton in PBS] and subsequently blocked with the blocking solution [2% (v/v) normal donkey serum (S30-100ML, Chemicon, Temecula, CA) in PBST] for 2 h. Then, sections were incubated with the primary antibody overnight at 4°C. Sections were washed with PBST three times and subsequently incubated with the secondary antibodies for 1 h at room temperature. Sections were then stained with 4′,6-diamidino-2-phenylindole, dihydrochloride (DAPI) (1 ng/ml; D1306, Thermo Fisher Scientific, Waltham, MA) in PBST for 30 min and washed twice with PBST. Stained sections were mounted with Fluoromount/Plus™ (K048, Diagnostic BioSystems, Pleasanton, CA, United States). Digital images were obtained using fluorescent microscopy systems [DeltaVision (Applied Precision Inc., Issaquah, WA, United States) equipped with CoolSNAP HQ2 camera (Photometrics, Tucson, AZ, United States), or Eclipse Ti-E (Nikon, Tokyo, Japan) equipped with an EMCCD camera (iXON DU-897, Andor Technology, Belfast, United Kingdom)], and confocal laser scanning microscopes [FLUOVIEW FV1000 (Olympus, Tokyo, Japan), or LSM900 (Carl Zeiss, Oberkochen, Germany)]. Digital images were analyzed using the Fiji ImageJ software (Schindelin et al., 2012) and ZEN (Zeiss). Immunohistochemical data were obtained from more than four different animals for each genotype, unless otherwise mentioned in the corresponding figure legends and table.

### Hematoxylin-eosin staining

Pancreatic tissue sections were subjected to HE staining using Mayer’s hematoxylin solution (131-09665, FUJIFILM Wako Pure Chemicals) and eosin Y solution (058-00062, FUJIFILM Wako). The images were observed using a microscope (ECLIPSEE800M, Nikon) and digital images were acquired using a digital slide scanner (NanoZoomer, Hamamatsu Photonics K.K., Shizuoka, Japan) and analyzed using the Fiji ImageJ software. HE staining data were obtained from more than four different animals for each genotype, unless otherwise mentioned in the corresponding figure legends and table.

### Azan staining

Pancreatic tissues were immersed in 10% (w/v) potassium dichromate solution containing 10% (w/v) trichloroacetic acid for 10 min, followed by a series of staining steps (accompanied by washing with water between steps) in Mallory azocarmine G solution (4,001-2, Muto Pure Chemicals Co., LTD., Tokyo, Japan) for 30 min, in 0.1% (v/v) aniline in 95% (v/v) ethanol (until nuclei stand out sharply), in 1% (v/v) acetic acid in 95% (v/v) ethanol for 1 min, in 5% (w/v) phosphotungstic acid solution for 1 h, and in Mallory aniline blue G solution (4,005-2, Muto Pure Chemicals) for 1 h. Digital images were acquired using the digital slide scanner NanoZoomer and analyzed using the Fiji ImageJ software. Azan staining data were obtained from more than four different animals for each genotype unless otherwise mentioned in the corresponding figure legends and table.

### TUNEL assay

Paraffin sections of the pancreas were used for cell death analysis using a TUNEL (TdT-mediated dUTP-X nick end labeling) staining kit (*In Situ* Cell Death Detection Kit, TMR red; 12156792910, Sigma-Aldrich) according to the manufacturer’s instructions. After TUNEL and DAPI staining, sections were mounted onto slides with Fluoromount, and digital images were acquired using a fluorescent microscopy system (DeltaVision) and analyzed using the Fiji ImageJ software.

### Amylase activity assay

Mice were deeply anesthetized with 2% isoflurane (099-06571, FUJIFILM Wako Pure Chemicals) and a vaporizer (SN-487-0T, SHINANO Manufacturing CO., LTD., Tokyo, Japan). Pancreatic tissues were dissected and homogenized by 15 stokes at 1,000 rpm in an ice-cold Teflon-glass homogenizer containing 0.5 ml of Amylase Assay Buffer (MAK009A, Sigma-Aldrich). Homogenates were centrifuged at 13,000 × *g* for 10 min at 4°C, and the resulting supernatant, designated pancreas protein extract sample, was subjected to amylase activity assay using the Amylase Activity Assay Kit (MAK009, Sigma-Aldrich) according to the manufacturer’s instructions. One unit (U) was defined as the amount of amylase that cleaves substrate ethylidene-pNP-G7 to generate 1.0 µmole of *p*-nitrophenol per min at 25°C.

### Electron microscopy

The fine architecture of the exocrine pancreas was analyzed *via* transmission electron microscopy (TEM) analysis of 2-month-old male *Cadps2* KO (*n* = 2) and WT (*n* = 2) mice. Under deep anesthesia with sodium pentobarbital, the animals were perfused transcardially with 0.1 M phosphate buffer (PB), pH 7.3, and subsequently with a mixture of 2.5% glutaraldehyde and 0.5% PFA in a 0.1 M phosphate buffer, pH 7.3. Dissected pancreatic tissues were fixed in the same fixative for 4 h followed by rinsing in 0.1 M PB three times for 30 min each. The fixed pancreatic tissue was cut into small pieces, approximately 1.5 mm in size, post-fixed in 1% OsO_4_ buffered at pH 7.2 with 0.1 M cacodylate for 2 h at 4°C, dehydrated through a graded ethanol series, and embedded in Epon-812 (Shell Chemical Co., Sewaren, NJ, United States). Ultrathin sections were prepared with a diamond knife (Diatom, Bienne, Switzerland) on an ultramicrotome (Reichert-Nissei, Tokyo, Japan). The tissue sections were examined using TEM (Hitachi H-7100 at 100 kV) after double staining with uranyl acetate and lead citrate. TEM analysis was also performed for 6-month-old male *Cadps2* KO (*n* = 3) and WT (*n* = 3) mice. Under deep anesthesia with sodium pentobarbital, the animals were perfused transcardially with 0.1 M phosphate buffer, pH 7.3, and subsequently with 4% PFA in a 0.1 M phosphate buffer, pH 7.3. Dissected pancreatic tissues were fixed in phosphate-buffered 2% glutaraldehyde (EM grade, Electron Microscopy Science, Hatfield, PA) in 0.1 M phosphate buffer at 4°C, and subsequently post-fixed in 2% osmium tetroxide (Crystal, Heraeus Chemicals, Port Elizabeth, South Africa) for 3 h in an ice bath. The specimens were dehydrated in a graded ethanol series and embedded in epoxy resin (at 60°C for 48 h). Ultrathin sections were obtained using an ultramicrotome as described above. Ultrathin sections were stained with 2% uranyl acetate in distilled water for 10 min and lead staining solution for 5 min and were subjected to TEM analysis (Hitachi H-7600 at 100 kV).

### Whole-exome sequencing, annotation, and *in silico* analysis

We conducted a mutational analysis of *CADPS2* in 186 unrelated Japanese patients with non-alcoholic CP *via* whole-exome sequencing as previously reported (Masamune et al., 2020). Genomic DNA was prepared from peripheral blood leukocytes and exon capture was performed using the SureSelectXT Human All Exon v5 capture kit (Agilent Technologies, Santa Clara, CA). We sequenced exon-enriched DNA libraries using the Illumina HiSeq 2,500 platform (Illumina, San Diego, CA). Paired-end 101-base pair reads were aligned to the reference human genome (UCSC hg19) using the Burrows-Wheeler Alignment tool 0.6.2 (http://bio-bwa.sourceforge.net/), and single nucleotide variants and insertions/deletions were annotated using ANNOVAR (BIOBASE, Wolfenbüttel, Germany; http://www.openbioinformatics.org/annovar/). The NM_001009571.3 GenBank reference sequence was used, and the variants were described according to the nomenclature recommended by the Human Genome Variation Society. We used SIFT (Sorting Intolerant From Tolerant; https://sift.bii.a-star.edu.sg/) and PolyPhen-2 (http://genetics.bwh.harvard.edu/pph2/) to predict the impact of an amino acid substitution on protein structure and function.

### Quantification and statistical analysis

The number of animals used in each experiment (N) and the analysis performed are specified in the figure legends. Data were analyzed using the two-tailed Student’s *t*-test. The data of the cytoplasmic distribution of SGs were analyzed using two-factor factorial analysis of variance (ANOVA) with a post-hoc test. Values are presented as the mean ± standard error of the mean (SEM). A *p*-value <0.05 was considered statistically significant. The tissue evaluation was performed by an investigator blinded to the mice genotypes. In the whole-exome analysis, the *CADPS2* variant frequencies in the general population in Japan (*n* = 8,380) were obtained from the Tohoku Medical Megabank database (https://jmorp.megabank.tohoku.ac.jp) (Tadaka et al., 2019). The significance of the differences in variant frequencies between patients and controls was tested using a two-sided Fisher’s exact test.

## Results

### Calcium-dependent activator protein for secretion 2 deficiency causes cellular injuries in the mouse pancreas

**FIGURE 1:**
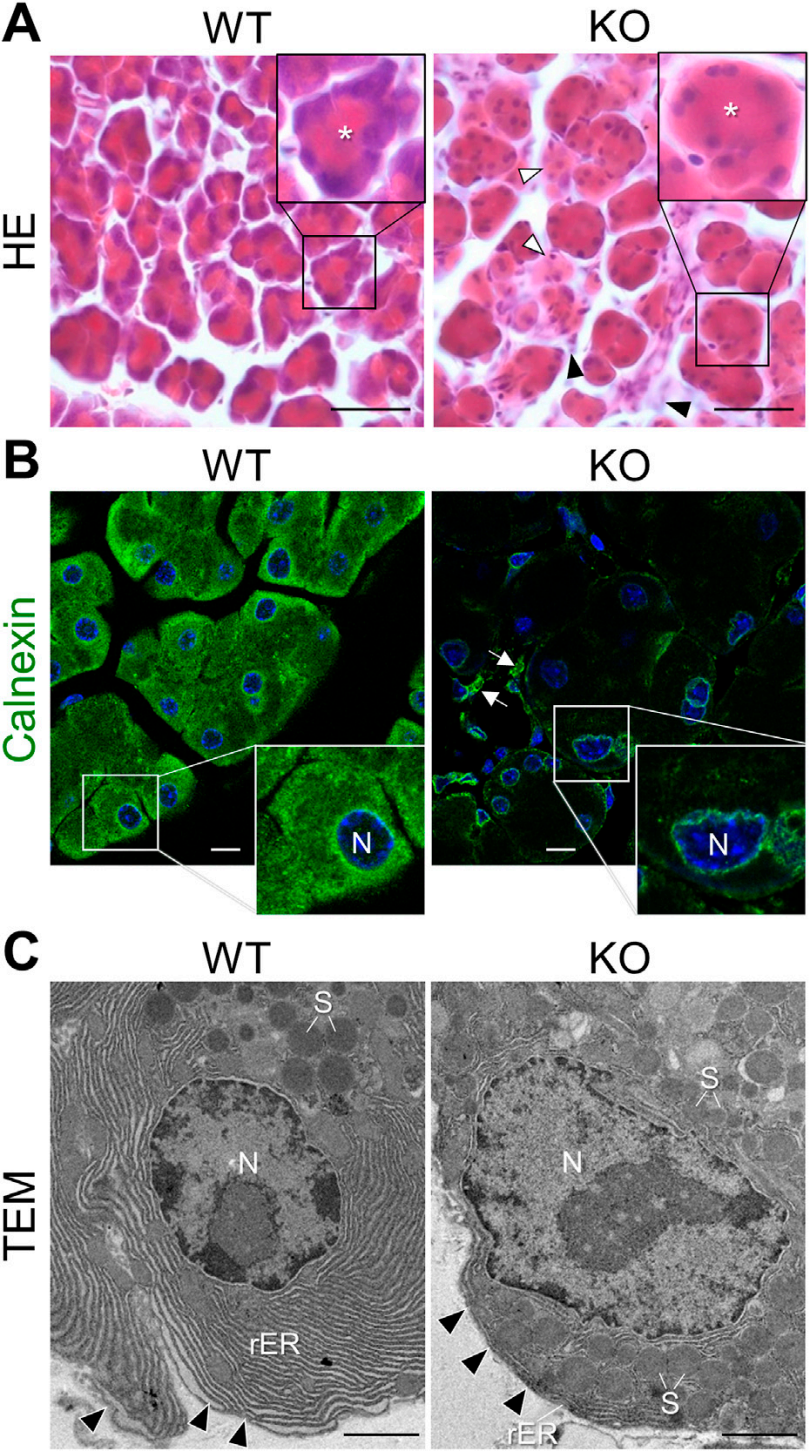
Cellular abnormalities in the exocrine pancreas of *Cadps2*-KO mice. **(A)**, HE-stained sections of the exocrine pancreas of WT and KO mice at 8 months of age. The inset in the top right corner shows a magnified view of acinus in each panel. *Asterisks* in the inset indicate the position of the acinar lumen. Acini of KO do not have sufficient hematoxylin-positive basophilic components compared to WT acini. In KO mice, *white arrowheads* indicate atrophic-like acini and *black arrowheads* indicate an increase in interstitial cells, such as pancreatic stellate cells and/or inflammatory cells. Scale bars: 25 μm. **(B)**, Immunostaining of the exocrine pancreas of WT and KO mice. Acinar cells were immunostained for the ER marker calnexin (*green*). DAPI-positive nuclei (*blue*). Inset in the bottom right shows a magnified view of the white square area shown in each panel. *Arrows* indicate interstitial cells immunopositive for calnexin. N, nucleus. Scale bars: 10 μm. **(C)**, Electron microscopy of the rough ER (rER) around the nucleus (N) in pancreatic acinar cells of WT and KO mice at 6 months of age. *Arrowheads* indicate the basal membrane. S, secretory granule. Scale bars: 1 μm.

There were no apparent pathological changes in the appearance of the pancreas in *Cadps2* **-**KO mice, except a reduction in the wet weight of KO mouse pancreas [at 4 months of age: WT (*n* = 3), 0.263 ± 0.017 mg; KO (*n* = 3), 0.183 ± 0.041 mg, Student’s *t*-test *p* = 0.035] and an abnormal coloration (slightly white) compared to the WT mouse pancreas (Supplementary Figure S1). However, histopathological analysis with HE staining revealed deterioration of acinar cell morphology with increased eosinophilic staining throughout the cytoplasm, and decreased hematoxylin-positive basophilic signals in the basolateral region in the KO exocrine pancreas compared to WT mice (Figure 1A). In addition, interstitial infiltration and atrophy-like features were observed in KO mice.

Basophilic hematoxylin signals in the basolateral cytoplasm of acinar cells highlighted the localization of the rough endoplasmic reticulum (rER), suggesting an abnormality in the rER network of acinar cells in *Cadps2*-KO mice. To address this possibility, we compared the rER between the acinar cells of KO and WT mice. In WT acinar cells, intense calnexin signals (a luminal protein of the ER) were observed around the nucleus and near the apical region, whereas in KO acinar cells, calnexin signals were severely reduced, except for weak signals around the nucleus (Figure 1B).

To further reveal the impact of CAPS2 loss on the rER, we analyzed the fine organellar architecture of acinar cells using TEM. WT mice showed highly ordered cisternae of the rER abundantly extended between the nucleus and basement membrane, while KO mice displayed considerable reduction and distortion of cisternae which was impeded by the presence of excessive amounts of SGs even around the nucleus (Figure 1C).

**FIGURE 2:**
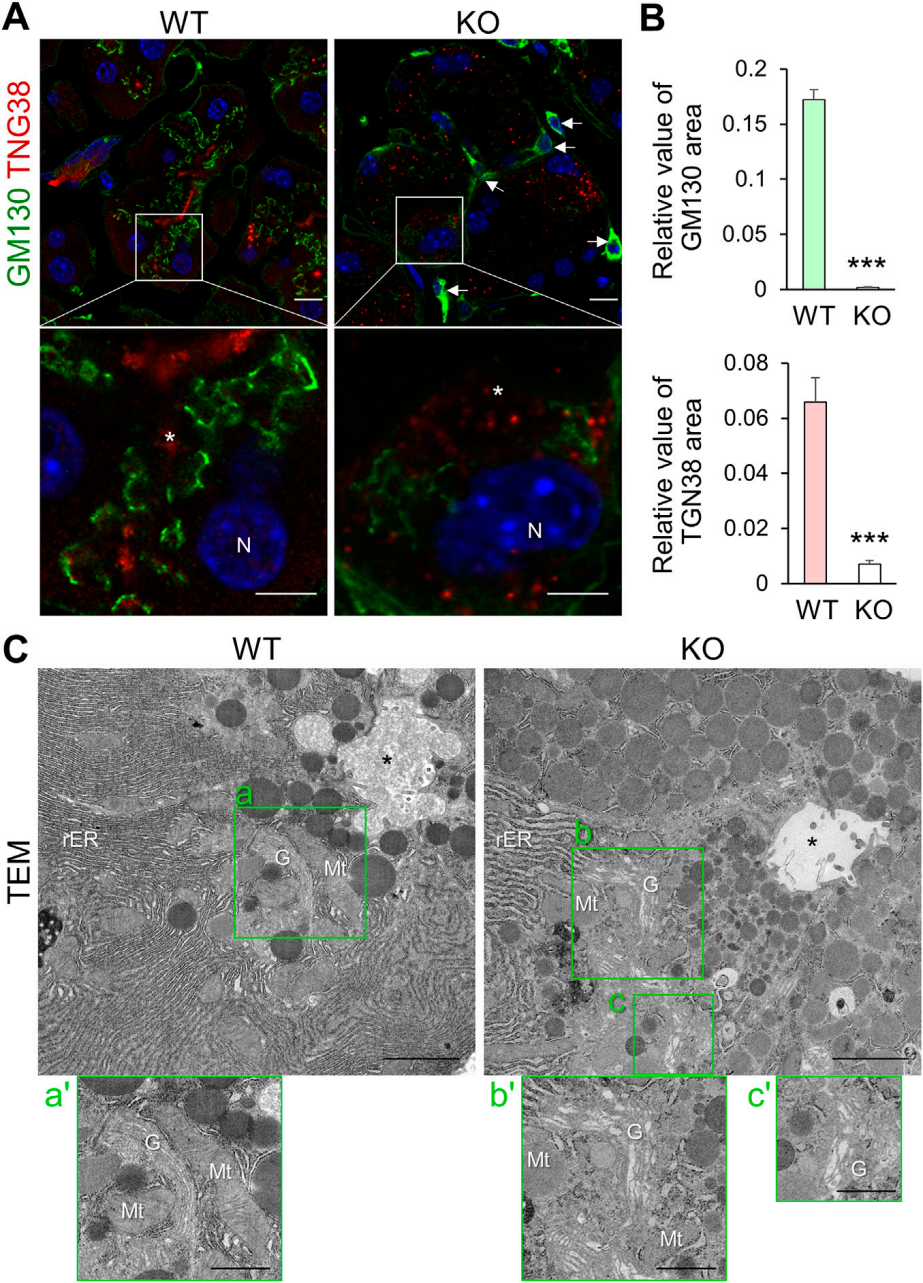
*Cadps2-*KO mice exhibit impaired organelle structure in the cytoplasm of pancreatic acinar cells. **(A)**, Immunostaining of pancreatic acinar cells of WT and KO mice. Acinar cells were double-stained with the *cis*-Golgi network maker GM130 (*green*) and the *trans*-Golgi network marker TGN38 (*red*). KO sections are typical acinar cells that still retained weak signals for TGN38 and GM130. DAPI-positive nuclei (*blue*). *Asterisks* indicate acinar lumen. *Arrows* indicate interstitial cells positive for GM130. N, nucleus. Scale bars: 10 μm in top panels and 5 μm in bottom magnified panels. **(B)**, Comparison of GM130-and TGN38-positive areas in pancreatic acinar cells between WT (*n* = 3) and KO (*n* = 4) mice at 6–9 months of age. In GM130 immunostaining, a total of 29 and 43 cells were analyzed for WT and KO, respectively. In TGN38-immunostaining, a total of 29 and 30 cells were analyzed for WT and KO, respectively. Relative values of immunopositive area/cell area (mean ± SEM, *p*-value determined using Student’s *t-*test): for GM130, WT = 0.1720 ± 0.0094, KO = 0.0012 ± 0.0004, *p* = 2.68E-33; for TGN38, WT = 0.0659 ± 0.0089, KO = 0.0071 ± 0.0013, *p* = 1.12E-08. ***p < 0.001. **(C)**, Electron microscopy of the fine architectures of the intracellular organelles in pancreatic acinar cells of WT and KO mice at 6 months of age. Images a’–c’ on the bottom of each panel are magnified views of the Golgi complex surrounded by green square areas a’–c’, respectively. *Asterisks* indicate acinar lumen. G, Golgi; Mt, mitochondria; N, nucleus; rER, rough ER. Scale bars: 1 μm for left parental images and 2 μm for right magnified images.

We next analyzed the Golgi apparatus and observed an irregular wavy pattern of GM130 signal (a *cis*-Golgi matrix protein) near the nucleus in the cytoplasm of WT acinar cells, which was severely reduced in those of KO mice (Figures 2A,B). TGN38 staining (an integral membrane protein of the TGN) was also reduced and severely dispersed and punctate in KO mice cells compared to WT mice (Figure 2A and Supplementary Figure S2). Moreover, TGN38 stained area in KO acinar cells, except for significantly atrophied cells, was reduced compared to that in WT cells (Figure 2B).

TGN38 cycles between the TGN and the plasma membrane but is localized predominantly in the TGN (Banting and Ponnambalam, 1997). The class II ARF small GTPases (ARF4 and ARF5) are mainly localized to the Golgi and also to other compartments along with the secretory pathway and is required for the retrograde transport of TGN38 from the recycling endosomes to the TGN (Nakai et al., 2013). We previously showed that CAPS2 interacts with ARF4/5 in the Golgi and CAPS2 knockdown affects the subcellular localization of ARF5 from the Golgi to a diffuse cytoplasmic pattern in αT3-1 cells, a mouse pituitary gonadotroph cell line (Sadakata et al., 2012a). Therefore, we analyzed their subcellular distribution in pancreatic acinar cells. Immunohistochemical analysis revealed dispersed ARF4 and ARF5 signals in KO cells compared to in WT cells (Supplementary Figure S3A), suggesting that loss-of-CAPS2 impairs Golgi localization of ARF4/5, which might affect the intracellular localization of TGN38.

Organellar markers themselves may have altered compartmental sorting or expression levels in KO cells. If the intracellular organellar structures and vesicular trafficking pathway are perturbed in KO cells, immunostaining with organellar markers may not reflect the actual morphology and cytoplasmic localization of the Golgi. Therefore, we analyzed the ER-Golgi structure using TEM and observed that KO acinar cells had dilated Golgi cisternae, as well as distended rER cisternae, compared to WT cells (Figure 2C). In addition, the Golgi complex in KO cells was distorted, and some parts appeared to be irregularly extended or dispersed within the cytoplasm in EM section images (Figure 2C-b’, c’).

Taken together, *Cadps2*-KO acinar cells had an impaired rER structure and Golgi network required for synthesizing and trafficking SGs and their contents, such as digestive enzymes.

### Excessive cytoplasmic accumulation of *α*-amylase and SGs in pancreatic acinar cells of *Cadps2*-knockout mice

**FIGURE 3:**
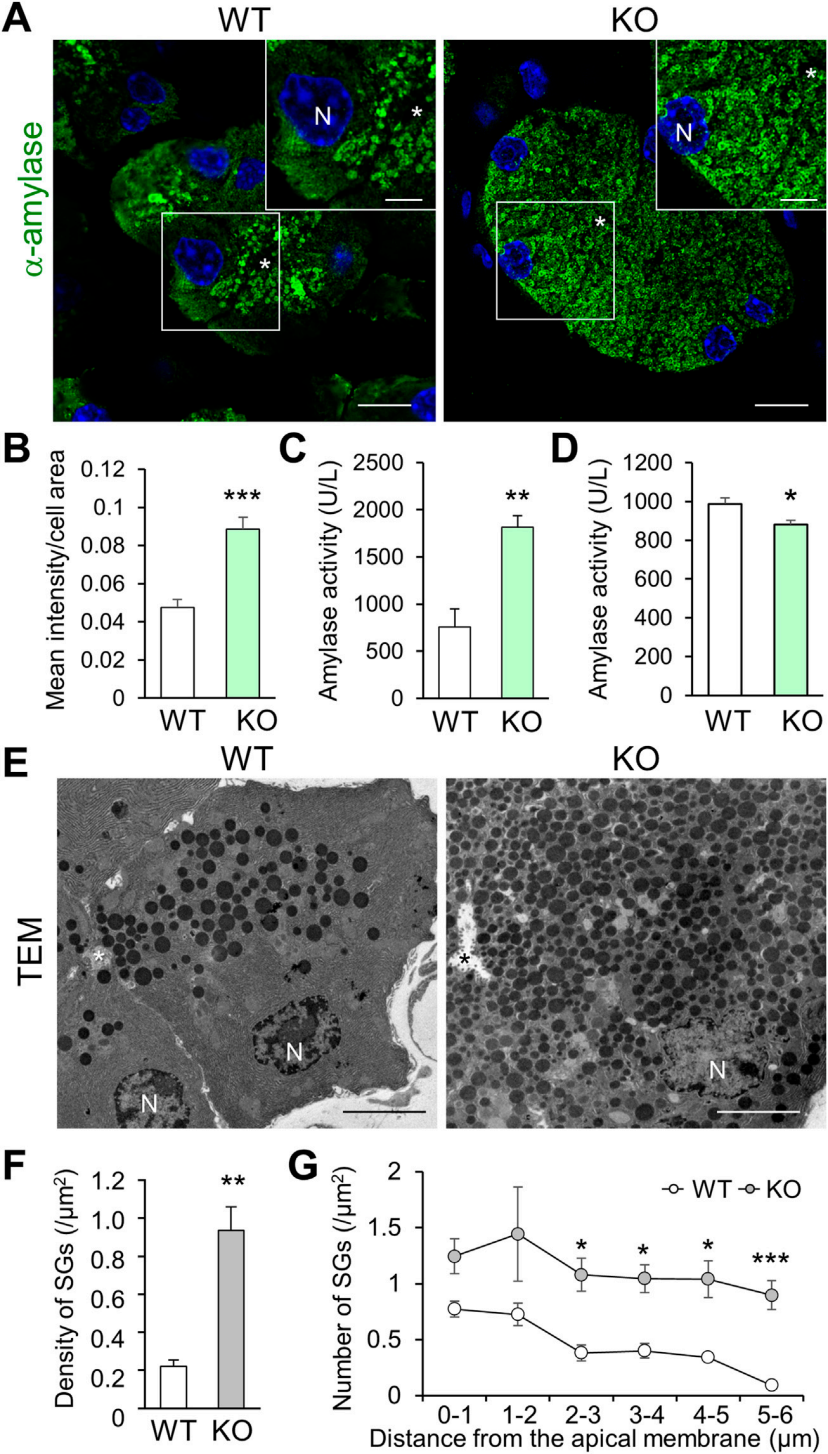
*Cadps2-*KO mice exhibit accumulation of α-amylase and secretory granules in the cytoplasm of pancreatic acinar cells. **(A)**, α-Amylase (*green*) immunohistochemistry in the exocrine pancreas of 1-month-old WT and KO mice. The inset in the top right corner represents a magnified image of the square area shown in each panel. *Asterisks*, the acinar lumen. N, DAPI (*blue*)-positive nucleus. Scale bars, 10 μm; 5 μm in the inset. **(B)**, Signal intensity of cytoplasmic α-amylase immunoreactivity. Ten different randomly chosen regions of interest (ROI) (127 × 127 μm) were analyzed for each genotype (WT: *n* = 5, KO: *n* = 4). Mean intensity/area ±SEM: WT, 0.048 ± 0.004; KO 0.089 ± 0.006. Student’s *t*-test: ***p = 0.00053. **(C,D)**, Enzymatic activity of α-amylase. One unit (U) was defined as the amount of amylase that cleaves substrate ethylidene-pNP-G7 to generate 1.0 µmole of *p*-nitrophenol per min at 25°C. **(C)**, Enzymatic activity of α-amylase in pancreatic protein extracts of 10-week-old WT and KO mice (U/L: mean ± SEM): WT (*n* = 3) 756.2 ± 122.2, KO (*n* = 3) 1815.1 ± 122.2. Student’s *t*-test: **p = 0.0095. **(D)**, Enzymatic activity of α-amylase in the serum of 11-week-old WT and KO mice (U/L: mean ± SEM): WT (*n* = 18) 987.6 ± 30.3, KO (*n* = 16) 881.1 ± 23.1. Student’s *t*-test: *p = 0.011. **(E)**, Electron micrograms of pancreatic acinar cells of 6-month-old WT and KO mice. *Asterisks*, the acinar lumen. N, nuclei. Scale bars: 5 μm. **(F,G)**, Intracellular distribution of SGs within the regions concentrically away from the acinar lumen. Electron micrograms of 19 and 18 cells from WT (*n* = 3) and KO (*n* = 3) mice, respectively, were analyzed. **(F)**, Mean density (SGs/μm^2^): WT 0.22 ± 0.03; KO 0.94 ± 0.12. Student’s *t*-test: *p = 0.0051. **(G)**, Density in acinar regions (SGs/μm^2^) within 1–5 μm concentric circles per 1 μm each away from the acinar membrane facing the lumen. The results of two-factor factorial ANOVA showed a statistical difference between genotypes (*F*_(1,24)_ = 52.74, *p* = 1.7 × 10^−7^), and also between distances (*F*_(5,24)_ = 3.73, *p* = 0.01), but not between genotype and distance (*F*_(5, 24)_ = 0.24, *p* = 0.94). Subsequent post-hoc Student’s *t*-test was performed between genotypes (values represent mean ± SEM): density <1 μm for WT 0.77 ± 0.07 and KO 1.25 ± 0.16, *p* = 0.051; 1–2 μm, WT 0.73 ± 0.10 and KO 1.44 ± 0.42, *p* = 0.172; 2–3 μm, WT 0.38 ± 0.07 and KO 1.08 ± 0.15, *p = 0.013; 3–4 μm, WT 0.40 ± 0.07 and KO 1.05 ± 0.12, *p = 0.010; 4–5 μm, WT 0.34 ± 0.04 and KO 1.04 ± 0.17, *p = 0.015; and 5–6 μm, WT 0.09 ± 0.03 and KO 0.90 ± 0.13, ***p = 0.004.

To examine the effect of abnormal rER and Golgi network on the subsequent trafficking of SGs, we analyzed cytoplasmic α-amylase (a luminal resident of SGs) in pancreatic acinar cells. *Cadps2*-KO mice exhibited increased cytoplasmic α-amylase immunoreactivity compared to WT mice, even at 1 month of age (Figures 3A,B), which progressively increased between 2 weeks and 8 months of age in KO mice (Supplementary Figure S4). Moreover, the amylase enzymatic activity increased in protein extracts of KO pancreas (Figure 3C), whereas it decreased in serum samples (extracellularly released amylase activity) compared to that of WT mice (Figure 3D). In addition, extracellular amylase released from primary pancreatic acini stimulated with secretagogue cholecystokinin-8 decreased in KO mice (Supplementary Figure S4), suggesting a deficit of amylase secretion in KO cells.

We next examined the intracellular distribution of SGs in the vicinity of pancreatic acinar lumen *via* TEM. Compared to that in WT cells, a higher number of SGs were abnormally accumulated within the cytoplasmic regions concentrically away from the acinar lumen in KO cells (Figure 3E). The total number of SGs was significantly increased in KO cells compared to in WT cells (Figure 3F). The significant accumulation of cytoplasmic SGs continued to the areas distant from the apical acinar membrane where exocytosis of SGs occurred (Figure 3G). The aberrant SG accumulation was detected in most KO cells that retained cell shape without strong atrophy and significantly extended from apical to basal cytoplasm (Supplementary Figure S5).

### Calcium-dependent activator protein for secretion 2 deficiency causes progressive deterioration of the fine intracellular architecture in pancreatic acinar cells

**FIGURE 4:**
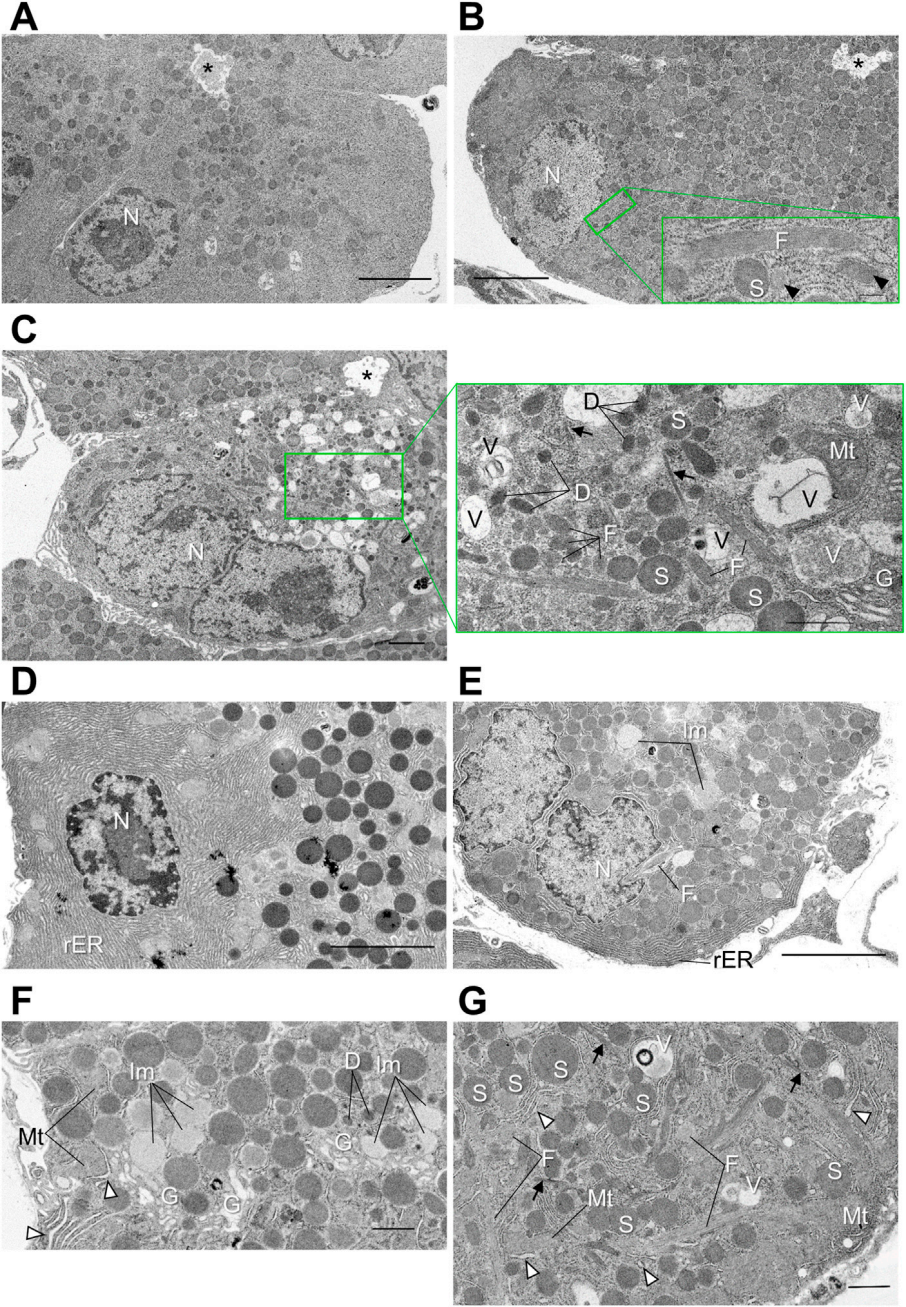
Distorted intracellular membrane structures in pancreatic acinar cells of *Cadps2*-KO mice. **(A–C)**, Electron micrographs of pancreatic acinar cells of WT **(A)** and KO **(B,C)** mice at 2 months of age. For KO mice, two types of acinar cells are shown: In **(B)**, cells at an early stage of atrophy and in **(C)**, atrophic KO cells with disorganized cytoarchitectures at the progressed stage. Compared to WT cells **(A)**, excessive amounts of SGs are accumulated in the cytoplasm of KO cells **(B)** from the apical site facing the lumen to the basal site where the nucleus resides. Image in the bottom right corner in panel B is a high magnification image of the green square containing a longitudinal section of a fibrillar granule. *Arrowheads* indicate transverse and oblique sections of fibrillar granules. In atrophic KO cells in **(C)**, round SGs are sparse. Image in the right of panel **(C)** is a high magnification image of the green square box. *Arrows* indicate fine bundles of intermediate filaments that are dispersed in the cytoplasm. *Asterisks* indicate acinar lumen. Scale bars: 5 μm in A and B; 200 nm in (b’); 2 μm in **(C)**; and 1 μm in (c’). **(D–G)**, Electron micrographs of pancreatic acinar cells of WT **(D)** and KO **(E–G)** mice at 6 months of age. Panels **(D,E)** show the basolateral area of WT and KO, respectively. Panel **(F)** is higher magnification image of the Golgi area. *White arrowheads* indicate distensions in rER lumina. Panel **(G)** is high magnification image of the basal section. *Arrows* indicate bundles of intermediate filaments. *White arrowheads* indicate distensions in rER lumina. D, dark vesicle; F, fibrillar granule; G, Golgi complex; Im, immature granule; Mt, mitochondria; N, nucleus; rER, rough ER; S, SG; V, vacuole. Scale bars: 5 μm in D and E; 1 μm in **(F)**; and 1 μm in **(G)**.

*Cadps2*-KO mice showed significant morphological changes in SG production- and trafficking-related organelles (rER and Golgi) in pancreatic acinar cells. To clarify the pathological feature in more detail, we further analyzed the progressive deterioration of the fine intracellular architecture of the KO mouse pancreas *via* TEM (Figure 4).

At 2 months of age (Figures 4A–C), the KO mouse pancreas was composed of heterogeneous acinar cells showing atrophic features from an early stage or relatively healthy-looking (Figure 4B) to the progressed stage with disordered cytoarchitecture (Figure 4C) compared to the WT mouse (Figure 4A). In addition to aberrantly accumulated SGs from apical to basal cytoplasm, KO cells (Figures 4B,C) contained elongated granules filled with fine longitudinal fibrils with a moderate electron density (Figures 4B,C), which were absent from WT cells (Figure 4A). Small, atrophic KO cells (Figure 4C), which were occasionally observed, contained the following unusual membrane structures: Dark vesicles that were relatively ellipsoidal; intermediate filaments that formed small bundles in the cytoplasm; vacuoles that contained diffuse materials at different densities; enclosed possible residues of membrane-bound organelles (Figure 4C). The cytoplasm of atrophic KO cells was also filled with different-sized granules with a low-medium density, as well as high-density mature SGs.

At 6 months of age (Figures 4D–G), the number of KO acinar cells with aberrant intracellular architectures increased compared to that at 2 months of age. The fibrillar granules, characteristic of KO cells but not WT cells (Figure 4D), were persistent after 6 months (Figures 4E,G). Meanwhile, some KO acinar cells continued to exhibit morphological characteristics indicative of continuous production of secretory proteins, including hypochromatic nuclei enclosing large nucleoli (Figure 4E) (but no significant change was noted in the nuclear size as shown in Supplementary Figure S6), a considerable amount of rER which penetrated labyrinthine spaces among SGs (Figures 4F,G), and well-developed Golgi complexes accompanied by immature granules of low electron density (Figure 4F). However, rER stacks were irregularly distended (Figures 4F,G).

Collectively, TEM analysis suggested chronic progressive signs of pancreatic acinar degeneration and atrophy, including the alteration and pleomorphism of the organelles and vesicular structures in *Cadps2*-KO mice.

### Calcium-dependent activator protein for secretion 2 deficiency induces progressive pancreatic acinar cell injury including fibrosis, lymphocytic infiltration, and apoptosis

**FIGURE 5:**
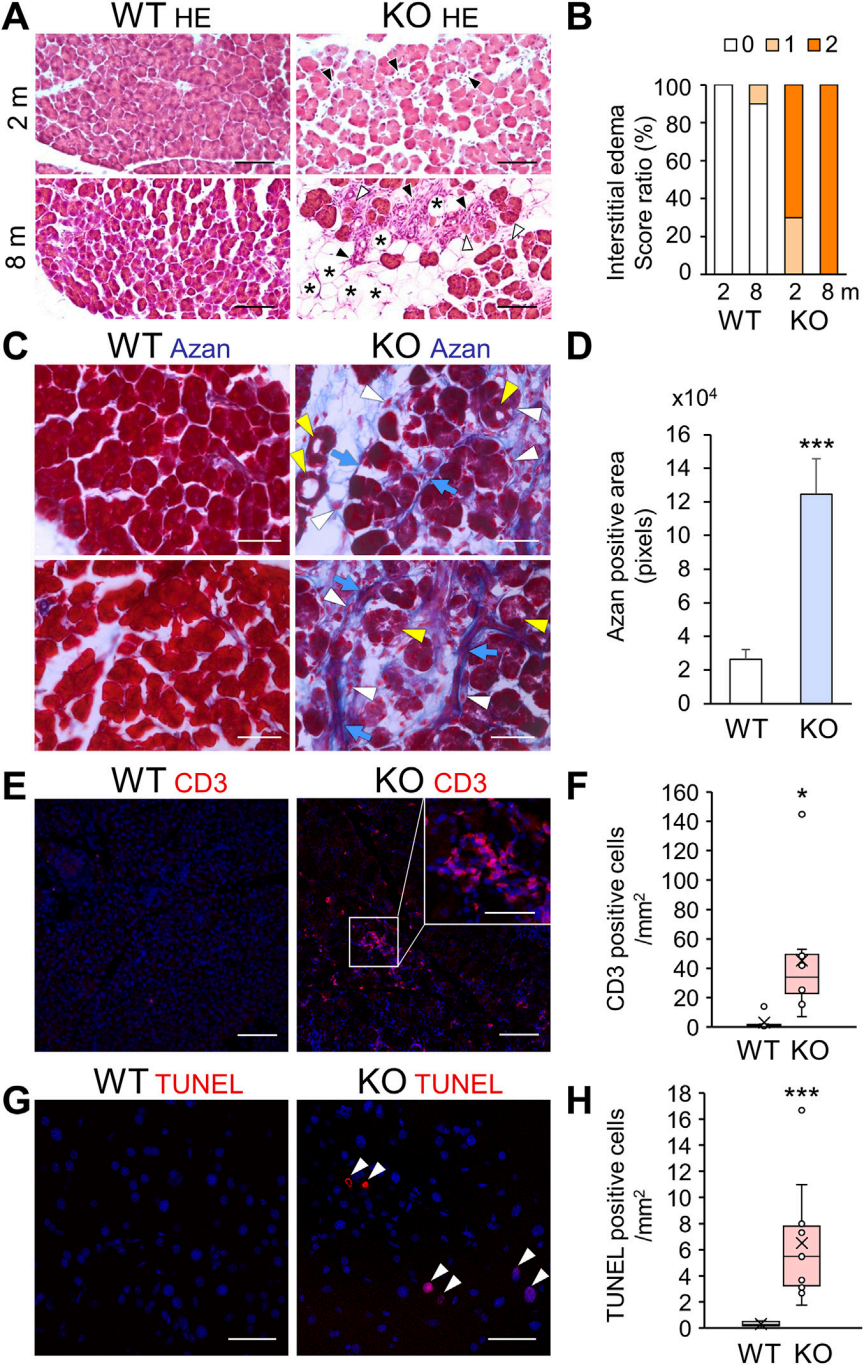
Acinar cell pathology with increased fibrosis, immune infiltration, and apoptosis in the exocrine pancreas of *Cadps2*-KO mice. **(A)**, HE-stained pancreatic sections of KO mice at 2 months (2 m, top row) and 8 months (8 m, bottom row) of age. Degeneration of acinar cells (*white arrowheads*), increase in adipocytes replacing acini (*asterisks*), and interstitial infiltration between acini (*black arrowheads*) of the exocrine pancreas are indicated. Scale bars: 100 μm. **(B)**, Score ratio (%) of interstitial edemas in the exocrine pancreas of WT and KO mice at 2 and 8 months (m) of age. The parameter was evaluated on a scale from 0 to 2 (0: normal or not detectable, 1: mild (<10% of visual field)] or focal accumulation, 2: moderate to severe (<10%) or diffusely scattered accumulation), as described previously (Barth et al., 2002; Geisz et al., 2019). Mice analyzed after 2 months, WT (*n* = 7) and KO (*n* = 10); after 8 months, WT (*n* = 10) and KO (*n* = 10). **(C)**, Azan (blue)-stained fibrotic collagen components in pancreatic sections of WT and KO mice at 2 months of age. *Blue arrows*, *white arrowheads,* and *yellow arrowheads* indicate fibrosis collagen, interstitial infiltration such as pancreatic stellate cells and/or inflammatory cells, and atrophic-like acinar cells, respectively. Scale bars: 100 μm. **(D)**, Azan-positive fibrosis areas (pixels). Ten ROI sections (100 × 220 μm) randomly selected for each pancreas sample of WT and KO mice at 2 months of age were analyzed [WT (*n* = 3) 26,338.9 ± 5,855.8 and KO (*n* = 7) 124,616.1 ± 21,124.3]. Values represent the mean ± SEM. Student’s *t*-test: ***p = 0.0022. **(E)**, CD3 (*red*) immunostaining analysis for the detection of T cell infiltration in pancreatic sections of WT and KO mice at 8–9 months of age. The inset in the top right corner in the KO image is an enlarged view of the CD3-positive cell cluster area indicated by a white square. DAPI staining (*blue*) for nuclei. Scale bars: 25 μm; 15 μm for inset image. **(F)**, CD3-positive inflammatory cells in WT and KO at 6–9 months of age [cells/mm^2^: average ±SEM: WT (n = 6) 1.8 ± 4.9 and KO (n = 8) 45.2 ± 40]. Student’s *t*-test: *p = 0.037. **(G)**, TUNEL-positive (*red*) apoptotic cells in pancreatic sections of 6-month-old WT and KO mice. DAPI staining (*blue*) for nuclei. Arrowheads show TUNEL-positive cells. Scale bars: 50 μm. **(H)**, TUNEL-positive cells/mm^2^ [mean ± SEM: WT (*n* = 9) 0.30 ± 0.17 and KO (*n* = 10) 6.51 ± 4.31]. Student’s *t*-test: ***p = 0.00076.

In addition to intracellular deterioration of pancreatic acinar cells, HE staining of *Cadps2*-KO mouse exocrine pancreas showed progressive acinar cell injury at 2 and 8 months of age with increased degenerated acini, adipocytes replacing acini, as well as interstitial infiltration between acini (Figure 5A). Progressive interstitial edema was also observed in KO mice compared to in WT mice (Figure 5B).

Considering the pathomorphological characteristics of the exocrine pancreas, we hypothesized that *Cadps2*-KO mice had congenital pancreatic anomalies, and thus we analyzed exocrine pancreatic cell injury, including fibrosis, lymphocyte infiltration, and cell death. Azan staining for collagen fibers revealed increased fibrosis (Figures 5C,D) in the vicinity of atrophic-like acini in KO mice (Figure 5C). The appearance of activated pancreatic stellate cells (aPSCs), which express α-smooth muscle actin (αSMA) and produce fibrous extracellular matrix including collagen (Hamada et al., 2022), was verified by the detection of increased αSMA-positive cells in KO pancreas (Supplementary Figure S3B). Immunostaining for CD3 (a T cell marker) exhibited increased CD3-positive inflammatory cell infiltration, in scattered or clustered patterns, near the acinar cells of KO mice compared to that of WT mice (Figures 5E,F). CD3-positive focal immune cell infiltrates were observed in degenerated areas (inset of Figure 5E). We used TUNEL analysis to determine the presence of apoptotic cells in the KO mouse pancreas since the secretory parenchyma was destroyed due to pancreatitis through necrosis/apoptosis. The KO mouse pancreas exhibited increased TUNEL-positive signals, in contrast to WT mouse pancreas, which infrequently exhibited TUNEL-positive signals (Figures 5G,H).

Taken together, these pathophysiological data suggested that *Cadps2*-KO mice had acinar cell injury affecting the exocrine pancreas.

### Pancreas-specific *Cadps2*-cKO mice exhibit similar pancreatic abnormalities as global knockout mice

**FIGURE 6:**
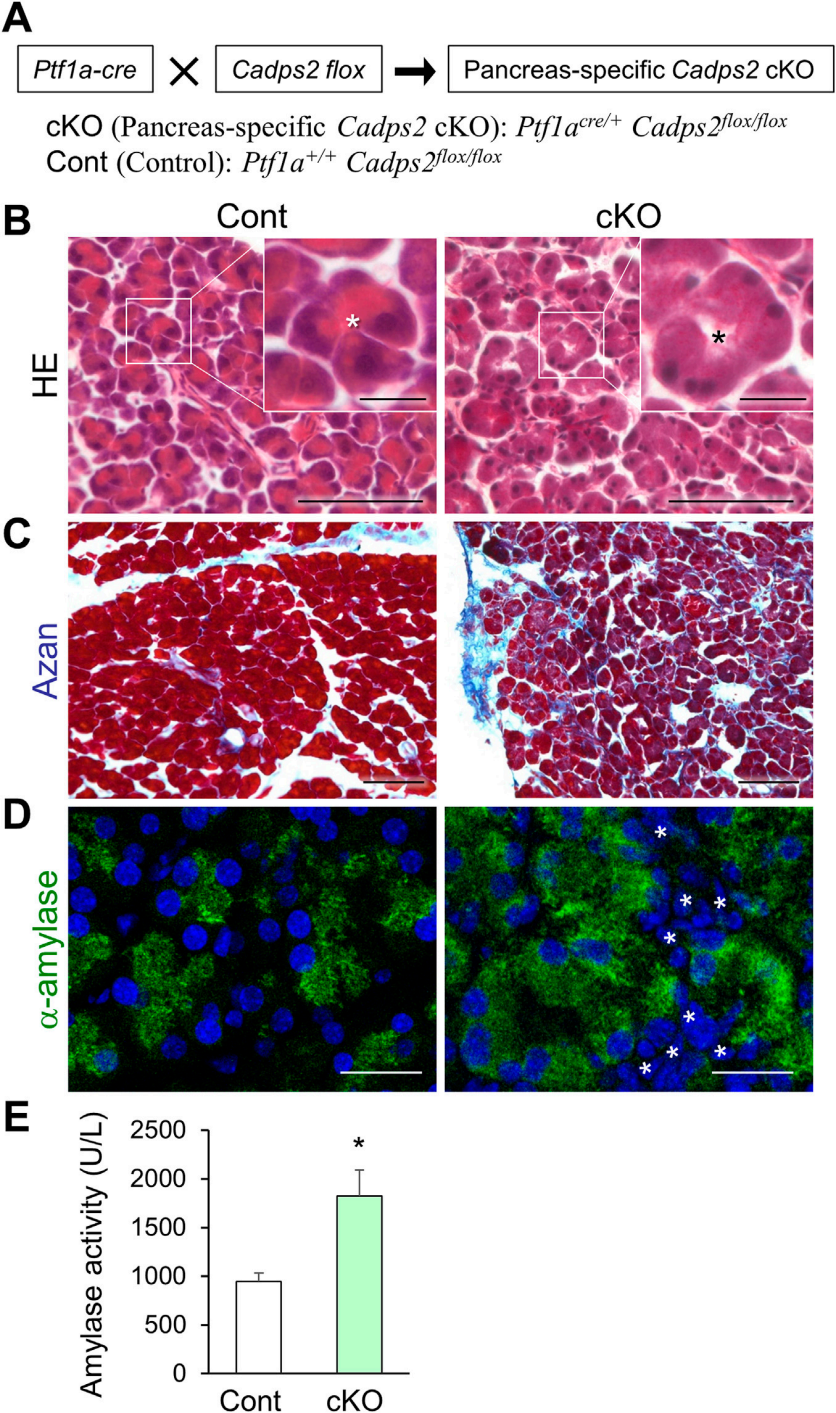
Pancreas-specific *Cadps2*-cKO mice showed similar cellular and biochemical phenotypes as *Cadps2*-KO mice. **(A)**, Pancreas-specific *Cadps2*-cKO (*Ptf1a* ^*cre/+*^ *Cadps2* ^*flox/flox*^) mice were generated by crossing *Cadps2* floxed mice (Fujima et al., 2021) with *Ptf1a*-driven *Cre* mice (Kawaguchi et al., 2002). *Ptf1a* ^*+/+*^ *Cadps2* ^*flox/flox*^ mice were used as the control (Cont). **(B)**, Reduced basophilic hematoxylin signals in the pancreatic acinar cells of *Cadps2*-cKO mice. HE-stained pancreatic sections of control and *Cadps2* cKO mice at 15 weeks of age. The inset in the top right corner represents magnified images of the area indicated by a square in each image. Asterisks indicate the acinar lumen. The basophilic hematoxylin signals in the basolateral cytoplasm of WT acinar cells were reduced in cKO mouse acinar cells. Scale bars: 100 μm; 20 μm for inset images. **(C)**, Increased fibrosis in the pancreas of cKO mice. Azan-stained fibrotic collagen components (*blue*) in the pancreatic sections of control and *Cadps2* cKO at 4 months of age. Scale bar: 100 μm. **(D)**, Increase in cytoplasmic amylase content and amylase enzyme activity in the pancreas of cKO mice. Immunohistochemistry for α-amylase (*green*) in the pancreatic acinar cells of control and cKO mice. DAPI staining (*blue*) for nuclei. *Asterisks* indicate an increased number of interstitial cells. Scale bars: 100 μm. **(E)**, Enzymatic activity of α-amylase in the pancreatic protein extracts of 10-week-old control and cKO mice (U/L; mean ± SEM: control (*n* = 4) 946.1 ± 87.9, cKO (n = 4) 1822.6 ± 270.6. Student’s *t*-test: *p = 0.022).

To further verify the direct impact of CAPS2 deficiency on pancreatic structure and function, we generated pancreas-specific *Cadps2*-cKO mice by crossing *Cadps2* floxed mice (Fujima et al., 2021) with the *Ptf1a-Cre* driver line (Kawaguchi et al., 2002) (Figure 6A) and analyzed their phenotypes. HE staining revealed reduced basolateral hematoxylin signals in the pancreatic acinar cells of pancreas-specific *Cadps2*-cKO mice (Figure 6B), similar to those of global *Cadps2*-KO mice (Figure 1A). Augmentation of azan-positive signals was also verified in the exocrine pancreas of cKO mice (Figure 6C). In addition, cKO mice exhibited increased cytoplasmic α-amylase immunoreactivity in the pancreatic acinar cells, as well as increased number of interstitial cells between acini (Figure 6D). Enhanced cellular amylase enzymatic activity was similarly detected in the pancreatic protein extracts of *Cadps2*-cKO mice compared to those of control mice (Figure 6E).

These pathophysiological abnormalities observed in pancreas-specific *Cadps2*-cKO mice suggested that CAPS2 deficiency in the pancreas was the primary cause of pancreatic acinar cell pathology in mice.

### Non-synonymous Calcium-dependent activator protein for secretion 2 variants occur in Japanese patients with non-alcoholic chronic pancreatitis

Finally, we sought to identify the CADPS2 genetic variation in patients with CP by conducting CADPS2 (chromosome 7, 7q31.32) mutational analysis in 186 Japanese patients with non-alcoholic CP *via* whole-exome sequencing and compared the distribution to that of 8,380 individuals from the general population in Japan (control subjects) (Tadaka et al., 2019; Tadaka et al., 2021). We identified four heterozygous, non-synonymous, missense CADPS2 variants in patients with non-alcoholic CP (Table 1). The p.Met224Val variant, which was deleterious based on the SIFT prediction, was identified in a patient with idiopathic CP who developed symptoms at 3 years of age. The patient did not have known pancreatitis-associated mutations, such as PRSS1, SPINK1, CTRC, CPA1, or TRPV6 (Hegyi et al., 2020; Masamune et al., 2020). This variant was absent in the control Japanese population and was disproportionally identified in patients with non-alcoholic CP compared to in the general population (*p* = 0.022). Meanwhile, the frequency of all other non-synonymous variants did not differ between patients with CP and controls.

**TABLE 1:** Non-synonymous *CADPS2* variants identified in Japanese patients with non-alcoholic CP.

Exon	Non-synonymous variant	Amino acid change	dbSNP	SIFT (score)	PolyPhen-2 (score)	CP (%)	Control^a^ (%)	*p*-Value^b^
1	c.107C>G	p.Pro36Arg	rs995154918	T (0.231)	P (0.783)	1/186 (0.5)	72 (0.9)	1.00
3	c.670A>G	p.Met224Val	rs942072846	D (0.039)	B (0)	1/186 (0.5)	0 (0)	0.022
26	c.3468G>C	p.Met1156Ile	rs749350918	T (0.296)	B (0.008)	1/186 (0.5)	4 (0.05)	0.10
28	c.3667C>T	p.His1223Tyr	rs766439346	T (1)	B (0)	1/186 (0.5)	14 (0.2)	0.28

B, benign; CP, chronic pancreatitis; D, deleterious; P, possibly damaging; SIFT, Sorting Intolerant From Tolerant; T, tolerated.

^a^ Control subjects: Whole genome reference panel 8.3KJPN (8,380 general Japanese individuals) (Tadaka et al., 2019; Tadaka et al., 2021) which has been deposited in the dbSNP of the NIH-NLB (https://www.ncbi.nlm.nih.gov/snp/rs671#frequency_tab).

^b^ *p*-values were calculated using a two-sided Fisher’s exact test (see Methods).

**FIGURE 7:**
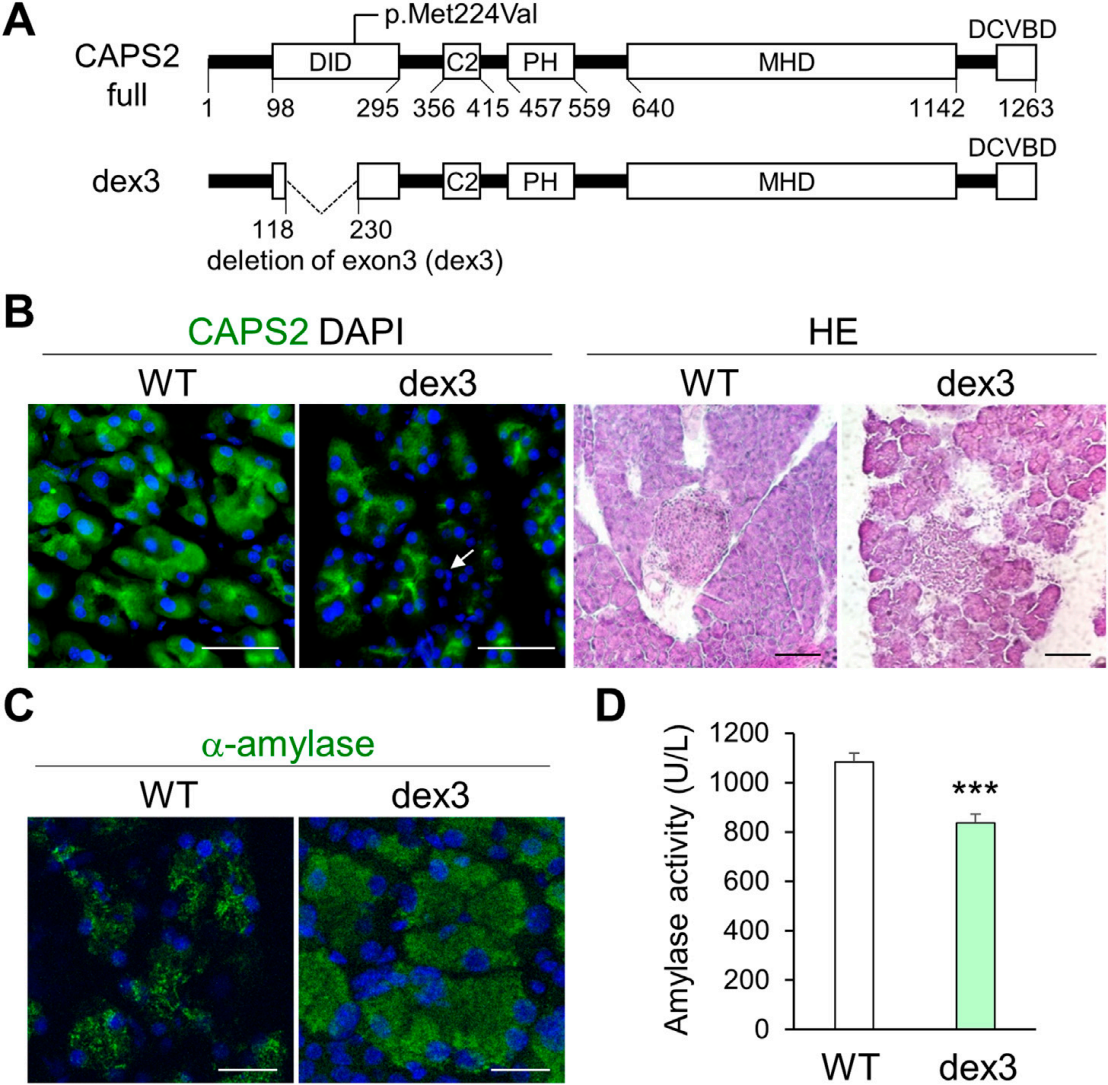
Pancreatic acinar cell degeneration and abnormal pancreatic exocrine function in mice with *Cadps2* exon3 (dex3) deletion. **(A)**, *Cadps2* gene structure of dex3 mice specifically expressing a rare alternative splicing variant *Cadps2-dex3* (deletion of exon 3) identified in patients with autism spectrum disorder. A human variant p. Met224Val identified in a patient with CP is mapped to exon3 which is located within the dynactin-interacting domain (DID). *Cadps2-dex3* mice have a deletion of exon3 in the *Cadps2* gene. Exon3 (119–229 aa); DID, dynactin-interacting domain; C2, C2 domain; PH, pleckstrin homology domain; MHD, Munc13 homology domain; DCVBD, dense-core vesicle binding domain. **(B)**, CAPS2-immunostaining (*green*) of pancreatic acini and HE-staining of pancreatic tissues in WT and *dex3* mice. DAPI staining (*blue*) for nuclei. An *arrow* indicates increased interstitial cells between pancreatic acini in *dex3* mice. Scale bars: 50 μm in immunostaining and 100 μm in HE-staining images. **(C)**, α-amylase (green) immunostaining images of WT and *dex3* mice. DAPI staining (*blue*) for nuclei. Scale bars: 25 μm. **(D)**, α-Amylase activity in the serum of 18-week-old WT and *Cadps2-dex3* mice (U/L; average ±SEM): WT (*n* = 10) 1084.07 ± 36.03, *dex3* (*n* = 10) 836.13 ± 35.69, Student’s *t*-test: *p = 0.0062.

The p.Met224Val variant is located within *CADPS2* exon3 (Figure 7A), which is involved in the intracellular localization of CAPS2 in neurons (Sadakata et al., 2007b; Sadakata et al., 2012b). Therefore, we analyzed the pancreatic phenotypes of *Cadps2-dex3* mice specifically expressing only a rare alternative splicing isoform with exon3 deletion (dex3) (Sadakata et al., 2012b). *Dex3* mice exhibited a slight change in CAPS2 localization and severe pancreatic acinar cell degeneration compared to WT mice (Figure 7B). Cytoplasmic amylase accumulation (Figure 7C) and decreased serum amylase activity (Figure 7D) were also observed in *dex3* mice similar to those in *Cadps2* KO mice (Figure 7), suggesting that the exon3-coded region is critical for proper functioning of CAPS2 in the exocrine pancreas.

## Discussion

CAPS is involved in TGN trafficking and/or SG exocytosis. In this study, we report that CAPS2 deficiency in mice resulted in various pathomorphological alterations in the intracellular membrane structures including the rER, Golgi, and SGs responsible for exocrine function in pancreatic acinar cells, as well as exocrine pancreatic cell injury including progressive acinar cell atrophy, increased αSMA-positive aPSCs leading to fibrosis, and inflammatory cell infiltration (Supplementary Figure S7). Pancreas-specific *Cadps2*-cKO mice exhibited similar pathological abnormalities in the exocrine pancreas as global *Cadps2-*KO mice. We also identified a rare variant of *CADPS2* in a patient with non-alcoholic CP. Our results suggest that CAPS2 plays a crucial role in the exocrine pancreas, and its deficiency may be associated with its congenital pathology.

We demonstrated pathomorphological abnormalities in the exocrine pancreas of *Cadps2*-KO mice including acinar cell atrophy, disturbances in the rER and Golgi network responsible for the synthesis and trafficking of SGs, excessive intracellular accumulation of SGs and their amylase content, and the appearance of aberrant vesicular structures, as well as fibrillar granules. Although further analyses for nuclear function are required, some KO cells had hypochromatic nuclei enclosing large nucleoli, suggesting that such cells may retain some functional activity.

The filamentous granules in *Cadps2* KO acinar cells correspond well to those which are generally known to be a characteristic of acinar cell carcinomas; both categories of granules display variously-elongated shapes and rich filaments bound in limiting membrane (for a review see (Chong et al., 1996)). These granules can transform into secretory granules, as indicated by the presence of their morphological intermediates in tumor tissues (Ordóñez and Mackay, 2000) and by transient appearance of the former, elongated granules in a certain phase of fetal development (Chong et al., 1996). The present study is the first demonstration of the filamentous granules in non-cancerous, mature acinar cells. Extensive studies will be required for elucidation of the nature of the granules and their involvement in physiology and pathology of the pancreas.

*Cadps2*-KO mouse pancreas tended to be a little whitish in appearance, and a similar color change was reported in the pancreas of *VAMP8*-KO (Wang et al., 2004) and *IRF2*-KO mice (Mashima et al., 2011), both of which revealed accumulation of pancreatic SGs. In addition, *Cadps2* KO mice exhibited pathophysiological phenotypes including a progressive loss of acini, alternatively increasing adipocytes and interstitial cells, leading to pancreatic acinar cell atrophy, fibrosis, and intense focal inflammation, which was reminiscent of that observed in a caerulein-induced pancreatitis mouse model (Nagashio et al., 2004). Similar phenotypes were observed in three independent *Cadps2-*modified mice, mainly global KO mice, pancreas-specific cKO, and *dex3* mice, suggesting that these pathological symptoms are primarily associated with CAPS2 function in the exocrine pancreas.

Acinar cell degeneration, fibrosis, and inflammation observed in the exocrine pancreas of *Cadps2*-KO mice were progressive but appeared relatively mild compared to those in chemically induced acute pancreatitis models and certain genetically-modified pancreatitis mouse models, many of which show fulminant symptoms (Merry and Petrov, 2018). Considering that CP in humans develops more than 10 years after symptom onset (Kleeff et al., 2017), *Cadps2*-KO mice may exhibit progressive pancreatic acinar cell pathology leading to CP-like outcomes. We also showed that KO mice exhibited an increase in TUNEL-positive signals in the pancreatic secretory parenchyma compared to WT, indicating apoptosis. It was reported that acinar cell death in pancreatitis is regulated by the balance between signaling mechanisms, i.e., “apoptosis” vs. “necrosis” (Mareninova et al., 2006). Necrotic cell death triggers an inflammatory process that contributes to pancreatic injury (Hoque and Mehal, 2015). From the appearance of atrophic-like cell injury under EM observation, a necrosis-like pathway probably represents a mechanism underlying acinar cell death in *Cadps2*-KO mice.

The CAPS family proteins tether and prime DCVs for Ca^2+^-triggered exocytosis in neurons and neuroendocrine cells (James and Martin, 2013; Messenger et al., 2014). Given the excessive intracellular accumulation of unsecreted α-amylase, as well as SGs, we hypothesized that *Cadps2*-KO acinar cells had a deficit in SG exocytosis. We have previously suggested another possible role of CAPS family proteins in the TGN-vesicle trafficking pathway since its deficiency in neurons perturbs the dense-core vesicle trafficking and Golgi structure, thereby decreasing the probability of neurotransmitter release (Sadakata et al., 2013). Our EM results revealed distorted and distended rER cisternae and swollen cisternae and disorganized stacks in the Golgi of *Cadps2*-KO pancreatic acinar cells. Immunostaining of the Golgi TGN marker, TGN38 (involved in protein sorting/targeting), rather than the CGN marker GM130 (involved in membrane tethering), exhibited a dispersed punctate pattern. These findings are similar to those reported for the amyloid-beta peptide (Aβ) transgenic mouse in which accumulation of Aβ leads to Golgi fragmentation (Joshi et al., 2014).

We suggest that loss of CAPS2 function may directly or indirectly affect TGN-vesicle trafficking as well as granule exocytosis through at least three possible cellular mechanisms: TGN38 puncta-associated inflammation, impairment of ARF4 localization to the TGN membrane, or an overloaded ER–Golgi pathway. We hypothesize that defects in any of these mechanisms could explain the pancreatic cell injury in KO mice.

*TGN38-positive puncta-associated inflammation*: TGN38 plays a role in protein sorting/targeting, post-TGN-vesicle formation, and TGN morphology maintenance, and cycles constitutively in compartments along the secretory pathway between the TGN and the plasma membrane but is localized predominantly to the TGN in its steady state (Banting and Ponnambalam, 1997). Interestingly, the dispersed TGN, which was detected by the formation of TGN38-positive puncta could act as a scaffold for the recruitment of the NOD-like receptors family pyrin domain-containing 3 (NLRP3) inflammasome (Nanda et al., 2021). Previous studies showed that the NLRP3 inflammasome is activated in pancreatic acinar cells of an acute pancreatitis mouse model and mediates pancreatic acinar cell death responses and systemic inflammation (Gao et al., 2021), and that the activity of NOD-like receptor NOD1 has a role in pancreatic inflammation in a CP mouse model (Watanabe et al., 2016). *Cadps2* KO acinar cells display infiltration of CD3-positive T cells together with fibrosis, caused by αSMA-positive aPSCs in response to injury or inflammation (Hamada et al., 2022), as described in caerulein-induced CP mice (Nagashio et al., 2004; Glaubitz et al., 2022). In addition, we hypothesize that NLRP3 inflammation pathway may be activated in *Cadps2* KO acinar cells with disperse TGN38-positive punctae.

### Lack of ARF4–Calcium-dependent activator protein for secretion 2 interaction in the *trans*-Golgi network

CAPS2 binds to the Golgi membrane and interacts with a class II ARF small GTPases ARF4 and ARF5 in a GDP-dependent manner (Sadakata et al., 2012a). ARF4 regulates protein trafficking in the early secretory pathway and is associated with Golgi stress (Reiling et al., 2013). *Arf4* knockdown inhibits the retrograde transport of TGN38 from the recycling endosomes to the TGN and increases localization of TGN38 in cytoplasmic punctate or tubular structures (Nakai et al., 2013). Here, we showed that the subcellular localization of both ARF4 and ARF5 changed to a diffuse pattern in the pancreatic acini of *Cadps2* KO mice, which may affect the localization of TGN38 to the intracellular compartments. Importantly, *Arf4-*KO mice show severe degeneration and fibrosis of the pancreas (Pearring et al., 2017). Therefore, pancreatic acinar cells lacking CAPS2 may be exposed to stress associated with vesicular trafficking, such as SG exocytosis, and subsequent endocytosis-recycling pathways, as well as Golgi membrane trafficking, possibly due to the loss of interaction between ARF4 and its Golgi-binding partner CAPS2.

### Overloaded ER–Golgi pathway

Our findings raise new questions, including whether the cytoplasmic accumulation of excessive amounts of unsecreted SGs affects the orderly distribution and function of other intracellular components (e.g., ER and/or Golgi), and whether the imbalance between the production of enzyme-carrying SGs and their material supply, which is likely due to over production of vesicle-associated proteins and membrane components with no recycling and endocytosis coupled with exocytosis, affects cell physiology, including proteostasis and lipidstasis. One potential theory is that an overwhelming load, beyond the capacity of the Golgi, causes impairment in protein modifications and vesicle trafficking due to insufficient Golgi functioning (Golgi stress) (Sasaki and Yoshida, 2019).

CP is a long-term progressive and irreversible inflammatory disorder causing morphological and functional damage to the exocrine pancreas, which greatly deteriorates the quality of life and decreases life expectancy (Kleeff et al., 2017), and the underlying mechanisms are not fully understood (Hegyi et al., 2020). The mutational analysis in 186 Japanese patients with non-alcoholic CP demonstrated that the p.Met224Val variant of *CADPS2* (chromosome 7, 7q31.32), which was absent in 8,380 control subjects, was overrepresented in CP. This variant was predicted to be deleterious based on SIFT, but not Polyphen-2. A study on correlations with primary and secondary structures showed that Met favors α-helix, while Val favors the β-strand (Malkov et al., 2008). A preliminary prediction suggests that the substitution of Met224 to Val may affect the secondary structure of the neighboring region. Although at this time, we were unable to provide evidence on the association between this missense substitution and CP, we are interested in the position of amino acid 224 within the coding region of exon 3 (Supplementary Figure S8), a region critical for intracellular trafficking of CAPS2, which was previously identified as deleted in patients with autism (Sadakata et al., 2007b). Mice expressing the exon 3-deleted CAPS2 isoform (dex3) showed a deficit in the axonal localization, thereby causing a disturbance in the secretion of neurotrophic factors from subcellular compartments (Sadakata et al., 2012b). Notably, *dex3* mice also exhibited similar pancreatic phenotypes as KO and cKO mice, suggesting a critical role of the exon3 region in CAPS2 protein function. However, further functional and validation studies in other populations, are warranted to clarify whether p.Met224Val affects pancreatic exocytosis.

In conclusion, we showed that CAPS2 is critical for the proper functioning of the exocrine pancreas, and its deficiency is associated with the congenital pathology of the exocrine pancreas in mice (Supplementary Figure S7). While our study demonstrates that CAPS2 deficiency in mice causes morphological and pathological changes in the exocrine pancreas, how CAPS2 regulates SG trafficking/exocytosis in pancreatic acinar cells remains to be determined, along with the role of CAPS2 in the human pancreas and the association of *CAPS2* variations with susceptibility to human pancreatic disorders. We believe that this study provides much-needed insight regarding the pancreatic exocrine regulation by CAPS2 as a potential risk factor for lifestyle-related pancreatic disease.

## Data Availability

The datasets presented in this study can be found in online repositories. The names of the repository/repositories and accession number(s) can be found below: https://www.ncbi.nlm.nih.gov/snp/rs671#frequency_tab NIH-NLM BioSample: https://www.ncbi.nlm.nih.gov/biosample/16789458. NIH NLM BioProject: https://www.ncbi.nlm.nih.gov/bioproject/PRJNA678214. https://jmorp.megabank.tohoku.ac.jp/202206.
