# Supplementary material for: Loss of CAPS2/Cadps2 leads to exocrine pancreatic cell injury and intracellular accumulation of secretory granules in mice
Source: Front Mol Biosci. 2022 Nov 7;9:1040237. doi: 10.3389/fmolb.2022.1040237 (PMC9676974; doi:10.3389/fmolb.2022.1040237)
Supplement: Supplementary file 1 [file DataSheet1.PDF]

Supplementary Material

1 Supplementary Data

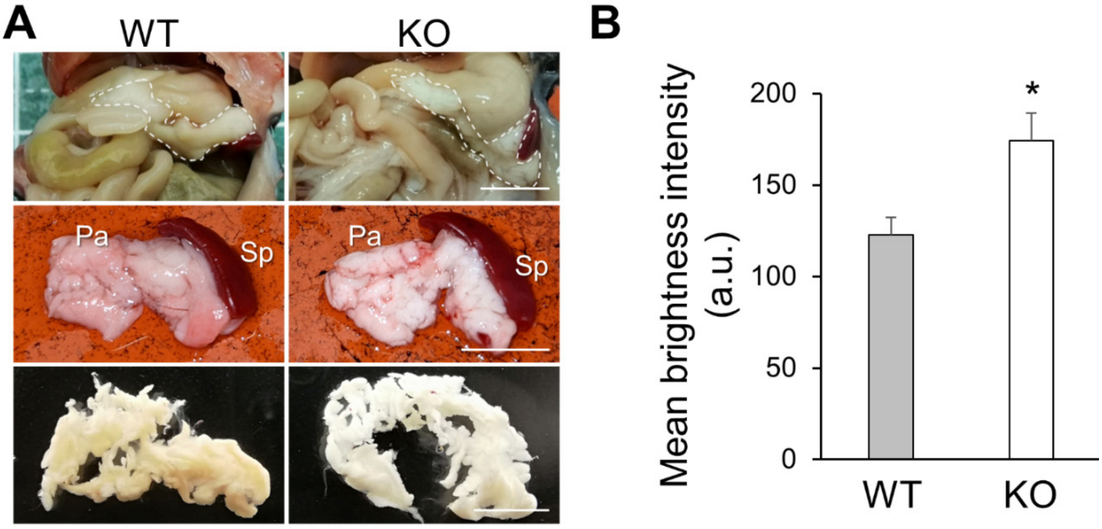

Supplementary Figure 1. The pancreas of WT and *Cadps2*-KO mice. A, Overall appearance of the pancreas in WT and KO mice at 2 months of age. Dashed lines in the top images outline the pancreas. Middle, fixed mouse samples. Bottom, un-fixed samples. Pa, pancreas; Sp, spleen. Scale bars: 1 cm. **B**, Mean signal intensity (a.u.) between WT and KO pancreas. The signal intensity of WT and KO pancreas grayscale images (six animals per genotype × three ROIs for each animal). Images of the KO pancreas are significantly brighter than those of WT pancreas (mean signal intensity ± SEM: KO = 174.24 ± 15.00, WT = 122.73 ± 9.45; $*p = 0.024$).

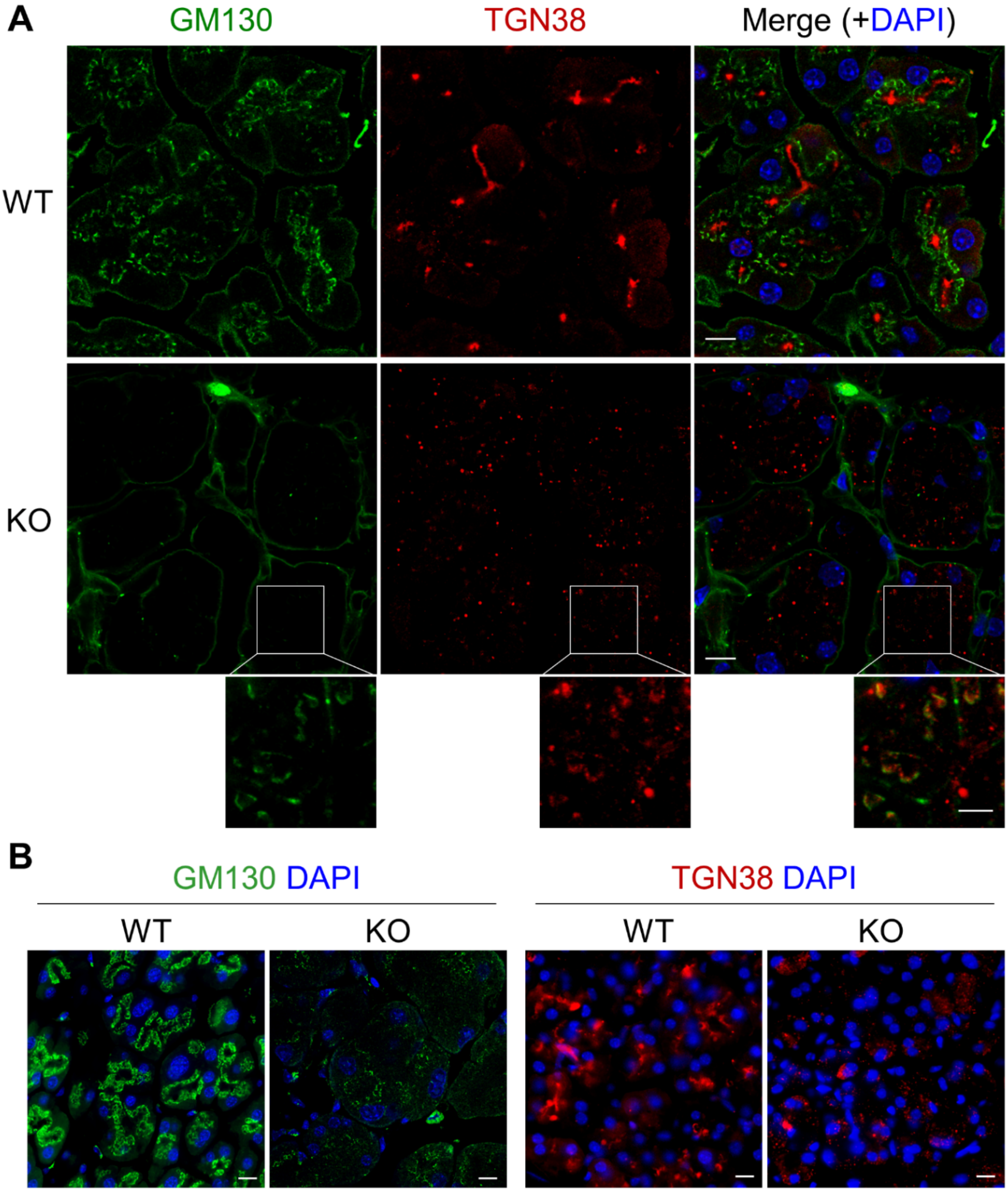

Supplementary Figure 2. Immunohistochemistry for Golgi protein markers in the pancreatic acini of WT and *Cadps2*-KO mice.

Immunostaining images **A** and **B** were acquired with a confocal fluorescent microscope and a conventional fluorescent microscope, respectively.

A, Double immunostaining for GM130 (*cis*-Golgi network [CGN] marker, *green*) and TGN38 (*trans*-Golgi network [TGN] marker, *red*). DAPI-positive nuclei (*blue*). The three panels in the bottom row represent magnified and "brightened" views of the white square areas indicated in the corresponding KO panels. In acinar cells of KO acini, the cytoplasmic immunosignals for both GM130 and TGN38

were reduced compared to those in WT. In the brightened views of KO acini, some extensions of GM130-positive CGN structures could be seen in the cytoplasm, whereas TGN38-positive TGN structures are considerably distorted or dispersed and even became punctate. Scale bars, 10 µm (5 µm in magnified images).
B, Single immunostaining for either GM130 (*green*) or TGN38 (*red*). DAPI-positive nuclei (*blue*) GM130-staining image in KO was carried out using the same section used for Calnexin staining in Fig. 2A. Scale bars, 10 µm.

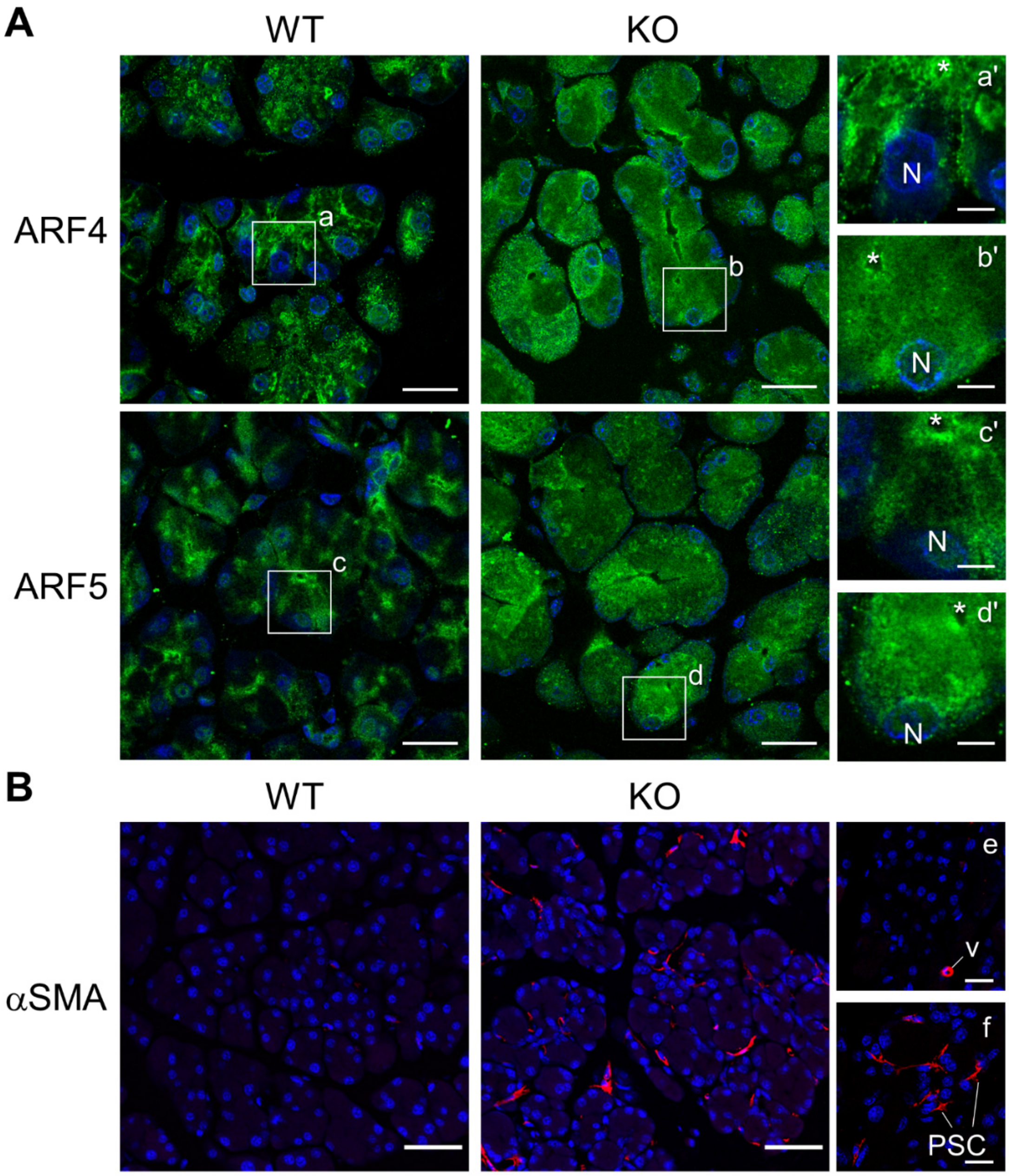

Supplementary Figure 3. Immunohistochemistry for the class II ARF small GTPase (ARF4 and ARF5) and αSMA in pancreatic acini of WT and *Cadps2*-KO mice.

A, Immunostaining for ARF4 and ARF5 (*green*) in WT and KO mice. Rabbit polyclonal anti-mouse ARF4 and ARF5 antibodies (Hosoi et al., 2019; Sadakata et al., 2010) and Alexa Flour 488 donkey anti-rabbit IgG (H+L) (1:5000, A-21206, Invitrogen) were used. DAPI-positive nuclei (*blue*). Images a'–d' in the right are magnified views of square areas a–d, respectively. In WT, ARF4 and ARF5 signals are mainly clustered in the vicinity of the apical and lateral membrane, with the integral membrane protein TGN38 localized mainly to the TGN (Figure 2A and Supplementary Figure 2A), whereas in KO the signals are dispersed in the cytoplasm compared to WT. *Asterisks* indicate acinar lumen. N, nucleus. Scale bars: 20 μm; 5 μm for images a'–d'.

B, Immunostaining for αSMA (*red*) in WT and KO mice. Mouse anti-actin, α-smooth muscle-Cy3 (1:500, C6198, Sigma Aldrich) were used. DAPI-positive nuclei (*blue*). Images e and f in the right show a vascular αSMA signal (v) in WT and pancreatic stellate cell (PSC) signals in KO. In KO, αSMA-positive activated PSCs are increased. Scale bars: 50 μm; 25 μm for images e and f.

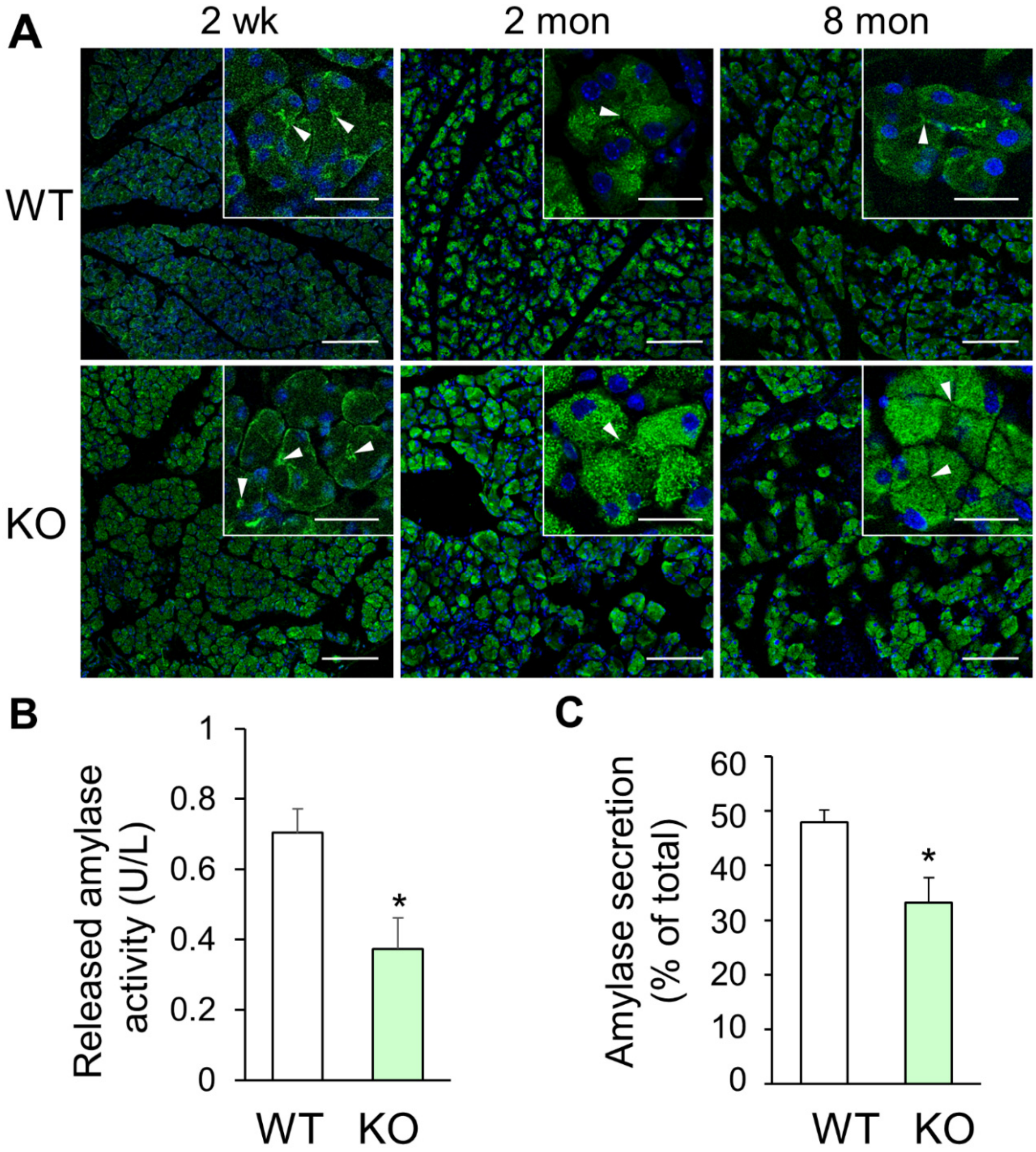

Supplementary Figure 4. Progressive accumulation of α-amylase immunoreactivity and reduction in secretagogue-induced amylase secretion in pancreatic acinar cells of *Cadps2*-KO mice.

A, Immunohistochemistry for α-amylase (green) in the pancreas of WT and KO mice at 2 weeks (wk), 2 months, and 8 months (mon) of age. Insets show magnified images. Arrowheads indicate the position of the acinar lumen. DAPI-positive nuclei (*blue*). Scale bars, 100 µm; for insets 20 µm.

B and **C**, Reduction in the enzymatic activity of amylase released from the organotypic cultures of the primary pancreatic acini of *Cadps2* KO mice in response to secretagogue CCK-8 stimulation. Primary pancreatic acini were isolated from WT and KO mice at 3–6 months of age.

B, Enzymatic activity of extracellular amylase (U/L) secreted into the culture media (average ± SEM): WT (n = 4) 0.703 ± 0.068, KO (n = 4) 0.374 ± 0.088. Student's *t*-test: $*p = 0.043$.

C, Percentage of amylase secretion per total amylase (sum of extracellular and cellular amylase) as shown below. Percentage (%) of total amylase; average ± SEM: WT (n = 4) 47.9 ± 2.3, KO (n = 4)

33.2 ± 4.6. Student's t-test: *p = 0.028. *Note*: the measured values of released/total amylase activities (secretion%) of four WT acini samples were 0.671/1.580 (52.45%), 0.927/2.011 (42.47%), 0.660/1.304 (46.10%), and 0.556/1.060 (50.61%); those of four KO acini samples were 0.125/0.545 (40.89%), 0.296/1.050 (22.94%), 0.571/1.396 (28.19%), and 0.503/1.230 (40.90%).

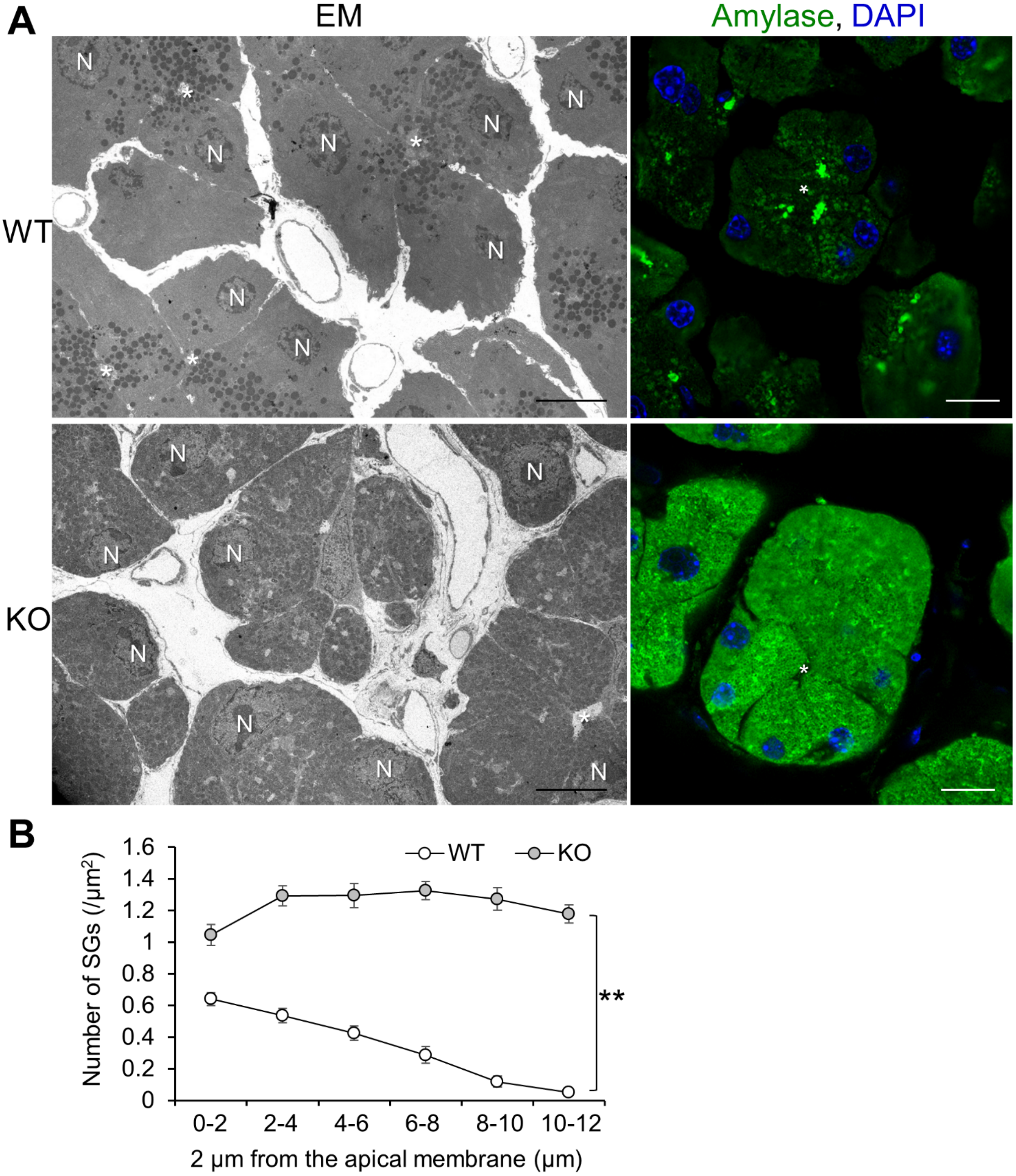

Supplementary Figure 5. Electron micrographs and amylase immunostaining images of WT and *Cadps2* KO pancreatic acini.

A, *Left*, electron micrographs; *right*, amylase (*green*) immunohistochemistry (DAPI, *blue*). In most acinar cells of WT acini, secretory granules are moderately diffused and distributed from the periphery of the acini lumen and the well-developed ER network surrounds the nucleus. By contrast, several acinar cells of KO acini display accumulated secretory granules throughout the cytoplasm

that display various degenerative signs (e.g., appearance of vacuoles and aberrant intracellular structures). *Asterisks* indicate acini lumen. N, nucleus. Scale bars, 10 µm.

B, Density of SGs (/µm^2) within the 2 µm region between the apical membrane to the basal cytoplasm (~0–12 µm) in WT and KO pancreatic acinar cells (WT cells n = 35, KO cells n = 25). For analysis of multiple parameters and conditions in this experiment, we employed repeated measures ANOVA tests, followed by *post-hoc* tests using the Tukey–Kramer test. As a result, repeated measures ANOVA showed a statistical significance of the interaction between genotype and distance ($F_{(5, 290)} = 28.29$, $p = 2.47E-23$). The Tukey–Kramer test showed significant difference in cytoplasmic spatial distribution of SGs between WT and KO mice (**$p < 0.01$). Data represent mean ± SEM/each distance: 0–2µm, WT 0.64 ± 0.04 and KO 1.05 ± 0.07; 2–4 µm, WT 0.54 ± 0.05 and KO 1.29 ± 0.06; 4–6 µm, WT 0.43 ± 0.05 and KO 1.30 ± 0.08; 6–8 µm, WT 0.29 ± 0.05 and KO 1.33 ± 0.06; 8–10 µm, WT 0.12 ± 0.03 and KO 1.27 ± 0.07; and 10–12 µm, WT 0.05 ± 0.02 and KO 1.18 ± 0.06.

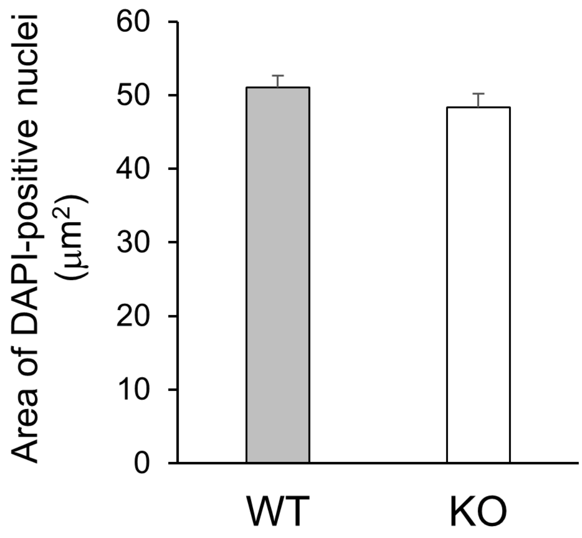

Supplementary Figure 6. Area of DAPI-stained nuclei in WT and *Cadps2* KO pancreatic acinar cells. Frozen sections of fixed pancreatic tissues were stained with DAPI. Area of DAPI-positive nuclei of acinar cells (excluding apparent apoptotic cells and interstitial cells with small nuclei) in images taken using a fluorescence microscope was measured using ImageJ (with the same threshold setting). Number of nuclei analyzed: WT n = 98, KO n = 89. Data represent mean ± SEM (μm^2): WT 51.04 ± 1.59, KO 48.37 ± 1.80. Student's t-test, $p = 0.133$.

Pancreatic acinar cell injury

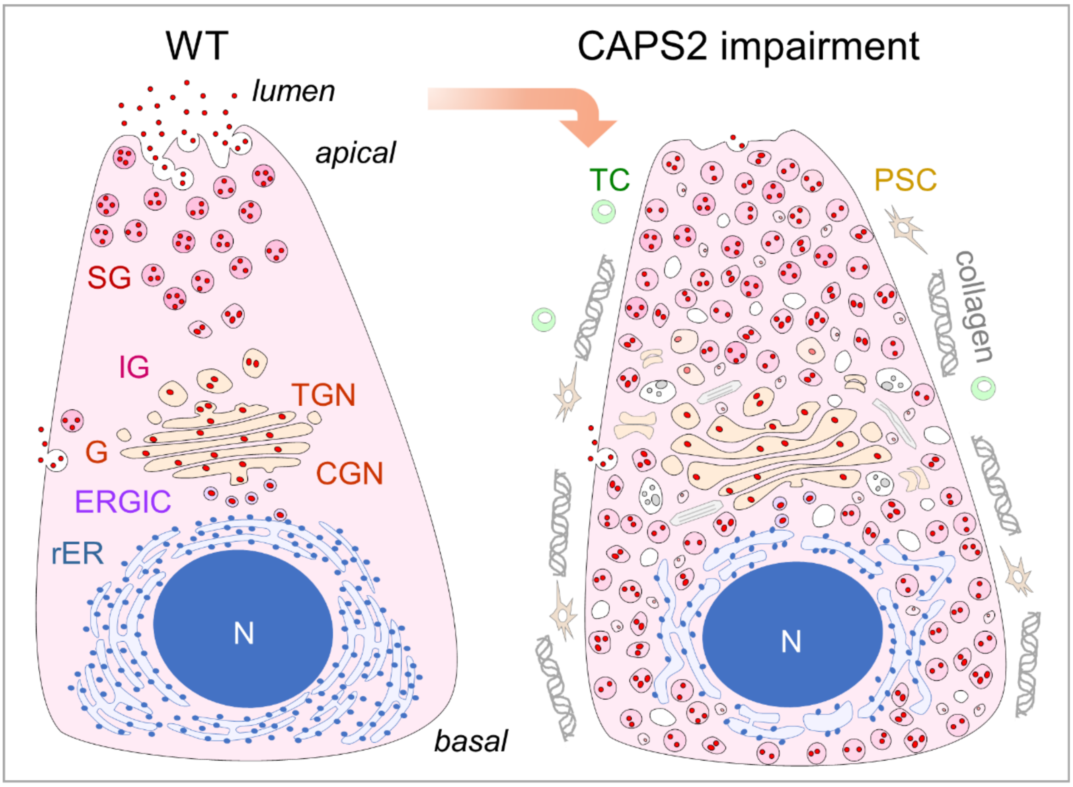

Pancreatic acinar cell injury

- Increased CD3-positive cells: T cell infiltration, inflammation
- Increased αSMA and collagen: PSC activation, fibrosis
- Increased TUNEL-positive cells: caspase-dependent apoptosis
- Appearance of aberrant intracellular structures (abnormal rER & Golgi, vacuoles w/wo membrane-like structures, filamentous granules, immature/small granules w/ low electron density, etc.)
- Appearance of atrophic acinar cells and adipocytes

Abnormal ER – Golgi trafficking pathway

- Decreased basophilic hematoxylin staining pattern
- Decreased/disorganized calnexin immunoreactivity
- Decreased/disorganized GM130 immunoreactivity
- Dispersed punctateTGN38 immunoreactivity
- Distorted/distended rER, dilated/extended Golgi

Abnormal pancreatic exocrine

- Accumulated cytoplasmic amylase immunoreactivity
- Accumulated cytoplasmic secretory (zymogen) granules
- Increased amylase activity in pancreatic protein extracts
- Decreased amylase activity in serum samples
- Decreased amylase secretion from primary acini

Supplementary Figure 7. CAPS2 function in exocrine pancreas. A schematic illustration of the results and implications. SG, secretory (zymogen) granule; IG, immature secretory granule; G, Golgi; CGN, cis-Golgi network; TGN, trans-Golgi network; rER, rough endoplasmic reticulum; ERGIC, ER–Golgi intermediate compartment; PSC, pancreatic stellate cell; TC, T cell; N, nucleus.

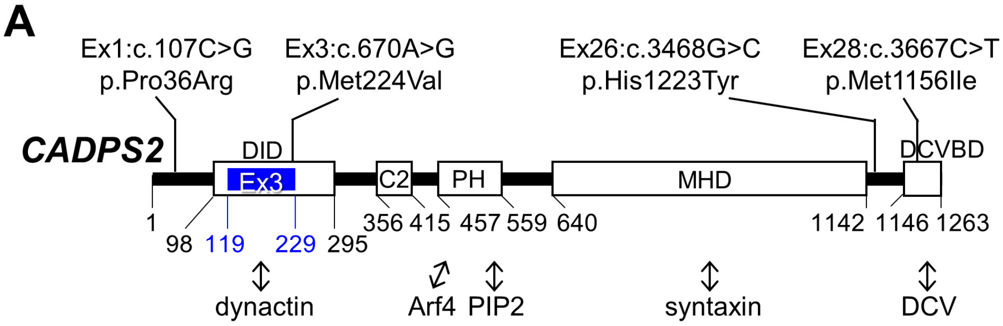

B

111 aa of the exon 3-coding region (119 – 229 aa)

Human Met-224 (ATG)

```
120       130       140       150       160
 |         |         |         |         |
LQLLKERFQAFLNGETQIVADEAFCNAVRSYYEVFLKSDRVA
hhhhhhhhhhhhttchheehhhhhhhhhhhhhhhhhhhhhhh
        170       180       190       200
         |         |         |         |
RMVQSGGCSANDFREVFKKNIEKRVRSLPEIDGLSKETVL
hhhhttcccchhhhhhhhhhhhhhhhcccchttcchhhhh
       210       220       229
         |         |         |
SSWIAKYDAIYRGEEDLCKQPNRMALSAVS
hhhhhhhhhhhttchhhhcccthhhhhhhh
```

Human Val-224 (GTG)

```
120       130       140       150       160
 |         |         |         |         |
LQLLKERFQAFLNGETQIVADEAFCNAVRSYYEVFLKSDRVA
hhhhhhhhhhhhcccchhhhhhhhhhhhhhhhhhhhhchhhh
        170       180       190       200
         |         |         |         |
RMVQSGGCSANDFREVFKKNIEKRVRSLPEIDGLSKETVL
hhhhcccccchhhhhhhhhhhhhhhhccccccccchhhhh
        210       220       229
         |         |         |
SSWIAKYDAIYRGEEDLCKQPNRVALSAVS
Hhhhhhhhhhhccccccccccccheehhhc
```

Supplementary Figure 8. Rare variant of CADPS2 identified in a patient with chronic pancreatitis and a comparison of the primary and predicted secondary structure of the exon 3-coding region between Met224 and Val224 variants of human *CADPS2*. A, Variants and functional domain structures of CAPS2/CADPS2. Locations of four variants identified are mapped. CAPS2 interacts with dynactin (a cofactor of the motor proteins dynein and kinesin II subtype), Arf4 (a class II Arf small GTPase involved in the vesicle trafficking and Golgi stress)/PIP_2

(phosphatidylinositol 4,5-bisphosphate), and Syntaxin (a SNARE protein involved in the membrane fusion during exocytosis) via DID, PH, and MHD, respectively (Grishanin et al., 2002; Sadakata et al., 2012; Sadakata et al., 2007). ex3, exon 3; DID, dynactin-interacting domain; C2, C2 domain; PH, pleckstrin homology domain; MHD, Munc13 homology domain; DCVBD, dense-core vesicle binding domain. **B**, 111 amino acid (aa) sequences between aa positions 119 and 229 encoded by exon 3 of the human *CADPS2* (chromosome 7, 7q31.32) are shown with the putative secondary structures predicted using the secondary structure prediction method (SOPM; https://npsa-prabi.ibcp.fr/cgi-bin/npsa_automat.pl?page=/NPSA/npsa_sopm.html) (Combet et al., 2000). The p.Met224Val position is indicated by red "M" and "V", respectively. Vertical lines show the featured region of the predicted secondary structures between Met224 and Val224 variants. h, a-helix; e, extended strand; t, β-turn; c, random coil.

2. Supplementary Methods

Amylase secretion assay

Preparation of pancreatic acinar cells: Mouse pancreatic acinar cells were prepared as previously described (Williams et al., 1978) with slight modification (Futatsugi et al., 2005; Kim et al., 2006). Briefly, mice were euthanized by cervical dislocation, and the pancreas was dissected and washed twice in bovine serum albumin (BSA)-containing buffer A (BSA+ buffer A) (buffer A: 140 mM NaCl, 5 mM KCl, 1 mM $MgCl_2$, 10 mM HEPES pH 7.4 adjusted with NaOH, 10 mM glucose, 0.1% BSA, 0.02% trypsin inhibitor from soybean [35543-04, Nacalai Tesque]). Exactly, 5 mL of the digestion solution (0.75 mg/mL collagenase Type V [038-17851, FUJIFILM Wako Pure Chemicals] in BSA+ buffer) was injected into different positions over the pancreatic parenchyma with a 27-gauge syringe (NN-2719S, Terumo) within 5 min, followed by incubation in a culture flask at 37 °C for 30 min with gentle shaking and repeated pipetting through a polypropylene pipette (with a 5-mm orifice) every 5 min. Digested pancreas solutions were transferred to a 50-mL centrifuge tube, mixed with 25 mL of BSA-lacking buffer A (BSA- buffer A), and then centrifuged at 100*g* for 3 min. After aspirating the supernatant, the pelleted cells were suspended in 30 mL of BSA- buffer A, and filtered through a nylon mesh cell strainer with a 100 µm pore (352360, Falcon, Corning Incorporated, Corning, NY).

CCK-8 stimulation: The prepared acinar cells (~10^6 cells) were re-centrifuged at 100*g* for 3 min and resuspended in 3 mL of Krebs-Ringer-HEPES (KRH) buffer (104 mM NaCl, 5 mM KCl, 1 mM KH_2PO_4, 1.2 mM $MgCl_2$, 25 mM HEPES-pH7.4, 2.5 mM D-glucose, GlutaMax [35050061, Invitrogen], MEM amino acids [132-15641, FUJIFILM Wako Pure Chemicals], MEM nonessential amino acids [06344-56, Nakali Tesque], 0.015% trypsin inhibitor from soybean, 0.2% BSA containing 100 pM of CCK-8 (CCK-Octapeptide 26-33, sulfated form [4100-v, Peptide Institute, Inc., Osaka, Japan]). Cell suspensions were incubated for 1 h at 37 °C with 5% CO_2. After incubation, the samples were centrifuged at 100 ×*g* for 3 min. Both the supernatant and the cell pellet were collected. The cell pellet was resuspended in 100 µL of RIPA buffer (50 mM Tris-HCl pH 7.4, 150 mM NaCl, 1% Triton X-100, 0.5% sodium deoxycholate, 0.1% SDS) and dissolved on ice for 30 min with gentle shaking at every 5 min. Both supernatants and cell extracts prepared from pancreatic acinar cells after CCK-8 stimulation were subsequently subjected to colorimetric analysis for amylase content.

Colorimetric analysis of amylase content: 1 mL of either the supernatant or the cell pallet extract was mixed with 1 mL of substrate solution (1% [w/v] soluble starch [S2004, Sigma-Aldrich] in phosphate buffer [20 mM sodium phosphate with 6.7 mM NaCl, pH 6.9]), and then incubated for 3 min at 20 °C. The reaction was terminated by adding 2 mL of dinitrosalicylic acid reagent (1% [w/v] 3,5-dinitrosalicylic acid [13525-82, Nacalai Tasque], 30% [w/v] sodium potassium (+)-tartrate tetrahydrate [Rochelle Salt; 31815-22, Nacalai Tasque], 20% 2 N NaOH) and then heating in boiling water for 5 min. After cooling down in running water, the reaction mixture was diluted by adding 20 mL of H_2O and mixing by inversion and dispended into two wells of 96-well plate in 100 µL aliquots each. The enzymatic activity was evaluated by measuring the absorbance at 540 nm using a plate reader (SpectraMax ABS Plus, Molecular Devices) and calculating the average value of two well samples. The amount of amylase released upon CCK-8 stimulation was determined via the percentage of the absorbance of the supernatant per that of the cell pellet extract.

3. Supplementary References

Combet, C., Blanchet, C., Geourjon, C., and Deléage, G. (2000). NPS@: network protein sequence analysis. *Trends in biochemical sciences* 25(3), 147-150. doi: 10.1016/s0968-0004(99)01540-6.

Futatsugi, A., Nakamura, T., Yamada, M.K., Ebisui, E., Nakamura, K., Uchida, K., et al. (2005). IP3 receptor types 2 and 3 mediate exocrine secretion underlying energy metabolism. *Science* 309(5744), 2232-2234. doi: 10.1126/science.1114110.

Grishanin, R.N., Klenchin, V.A., Loyet, K.M., Kowalchyk, J.A., Ann, K., and Martin, T.F. (2002). Membrane association domains in Ca2+-dependent activator protein for secretion mediate plasma membrane and dense-core vesicle binding required for Ca2+-dependent exocytosis. *J Biol Chem* 277(24), 22025-22034. doi: 10.1074/jbc.M201614200.

Hosoi, N., Shibasaki, K., Hosono, M., Konno, A., Shinoda, Y., Kiyonari, H., et al. (2019). Deletion of Class II ADP-Ribosylation Factors in Mice Causes Tremor by the Nav1.6 Loss in Cerebellar Purkinje Cell Axon Initial Segments. *J Neurosci* 39(32), 6339-6353. doi: 10.1523/jneurosci.2002-18.2019.

Kim, J.Y., Zeng, W., Kiselyov, K., Yuan, J.P., Dehoff, M.H., Mikoshiba, K., et al. (2006). Homer 1 mediates store- and inositol 1,4,5-trisphosphate receptor-dependent translocation and retrieval of TRPC3 to the plasma membrane. *J Biol Chem* 281(43), 32540-32549. doi: 10.1074/jbc.M602496200.

Sadakata, T., Itakura, M., Kozaki, S., Sekine, Y., Takahashi, M., and Furuichi, T. (2006). Differential distributions of the Ca2+ -dependent activator protein for secretion family proteins (CAPS2 and CAPS1) in the mouse brain. *J Comp Neurol* 495(6), 735-753. doi: 10.1002/cne.20947.

Sadakata, T., Mizoguchi, A., Sato, Y., Katoh-Semba, R., Fukuda, M., Mikoshiba, K., et al. (2004). The secretory granule-associated protein CAPS2 regulates neurotrophin release and cell survival. *J Neurosci* 24(1), 43-52. doi: 10.1523/jneurosci.2528-03.2004.

Sadakata, T., Sekine, Y., Oka, M., Itakura, M., Takahashi, M., and Furuichi, T. (2012). Calcium-dependent activator protein for secretion 2 interacts with the class II ARF small GTPases and regulates dense-core vesicle trafficking. *Febs j* 279(3), 384-394. doi: 10.1111/j.1742-4658.2011.08431.x.

Sadakata, T., Shinoda, Y., Sekine, Y., Saruta, C., Itakura, M., Takahashi, M., et al. (2010). Interaction of calcium-dependent activator protein for secretion 1 (CAPS1) with the class II ADP-ribosylation factor small GTPases is required for dense-core vesicle trafficking in the trans-Golgi network. *J Biol Chem* 285(49), 38710-38719. doi: 10.1074/jbc.M110.137414.

Sadakata, T., Washida, M., Iwayama, Y., Shoji, S., Sato, Y., Ohkura, T., et al. (2007). Autistic-like phenotypes in Cadps2-knockout mice and aberrant CADPS2 splicing in autistic patients. *The Journal of clinical investigation* 117(4), 931-943. doi: 10.1172/jci29031.

Williams, J.A., Korc, M., and Dormer, R.L. (1978). Action of secretagogues on a new preparation of functionally intact, isolated pancreatic acini. *The American journal of physiology* 235(5), 517-524. doi: 10.1152/ajpendo.1978.235.5.E517.
